# The Picornavirus Precursor 3CD Has Different Conformational Dynamics Compared to 3C^pro^ and 3D^pol^ in Functionally Relevant Regions

**DOI:** 10.3390/v13030442

**Published:** 2021-03-09

**Authors:** Dennis S. Winston, David D. Boehr

**Affiliations:** Department of Chemistry, The Pennsylvania State University, University Park, PA 16802, USA; dxw343@psu.edu

**Keywords:** picornavirus, poliovirus, polyprotein, virus precursor protein, protease, polymerase, protein dynamics, allostery, NMR

## Abstract

Viruses have evolved numerous strategies to maximize the use of their limited genetic material, including proteolytic cleavage of polyproteins to yield products with different functions. The poliovirus polyprotein 3CD is involved in important protein-protein, protein-RNA and protein-lipid interactions in viral replication and infection. It is a precursor to the 3C protease and 3D RNA-dependent RNA polymerase, but has different protease specificity, is not an active polymerase, and participates in other interactions differently than its processed products. These functional differences are poorly explained by the known X-ray crystal structures. It has been proposed that functional differences might be due to differences in conformational dynamics between 3C, 3D and 3CD. To address this possibility, we conducted nuclear magnetic resonance spectroscopy experiments, including multiple quantum relaxation dispersion, chemical exchange saturation transfer and methyl spin-spin relaxation, to probe conformational dynamics across multiple timescales. Indeed, these studies identified differences in conformational dynamics in functionally important regions, including enzyme active sites, and RNA and lipid binding sites. Expansion of the conformational ensemble available to 3CD may allow it to perform additional functions not observed in 3C and 3D alone despite having nearly identical lowest-energy structures.

## 1. Introduction

RNA viruses need to produce proteins that help evade the host immune defense, allow the virus to replicate, and establish new infections [1,2,3]. A typical positive-strand RNA virus codes for less than a dozen proteins; poliovirus, studied here, codes for ten proteins after proteolytic processing, four of which make up the capsid [4,5]. Positive-strand RNA viruses have evolved strategies for maximizing the use of their limited coding capacity. One strategy, which we will focus on here, is to encode a polyprotein translated from one large open reading frame, which is then cleaved by virally encoded proteases to yield individual proteins with unique functions and precursor intermediates that may have different functions than the fully processed products [1,6,7]. An important example is the poliovirus 3CD protein, a key protein regulating the spatial and temporal control of virus replication through its interactions with replication membranes, virus RNA and virus and host proteins [8,9,10,11,12,13]. 3CD is also proteolytically processed to yield separate 3C and 3D proteins, which play distinct functions from those of 3CD in virus replication [9,10,11,12,13,14,15,16,17]. As the 3C and 3D proteins are nearly structurally identical to their domain counterparts in 3CD, how different functions arise between the precursor and fully processed proteins has remained unclear [5,8]. It has been proposed that conformational dynamics, that is, fluctuations away from the lowest energy conformations represented by the X-ray crystal structures, may be different between 3C, 3D and their domain counterparts in 3CD, thereby driving their different functions [18]. Indeed, we show in this manuscript that there are different conformational dynamics between 3C/3D and 3CD across multiple timescales, which may contribute to their different functions.

**Figure 1:**
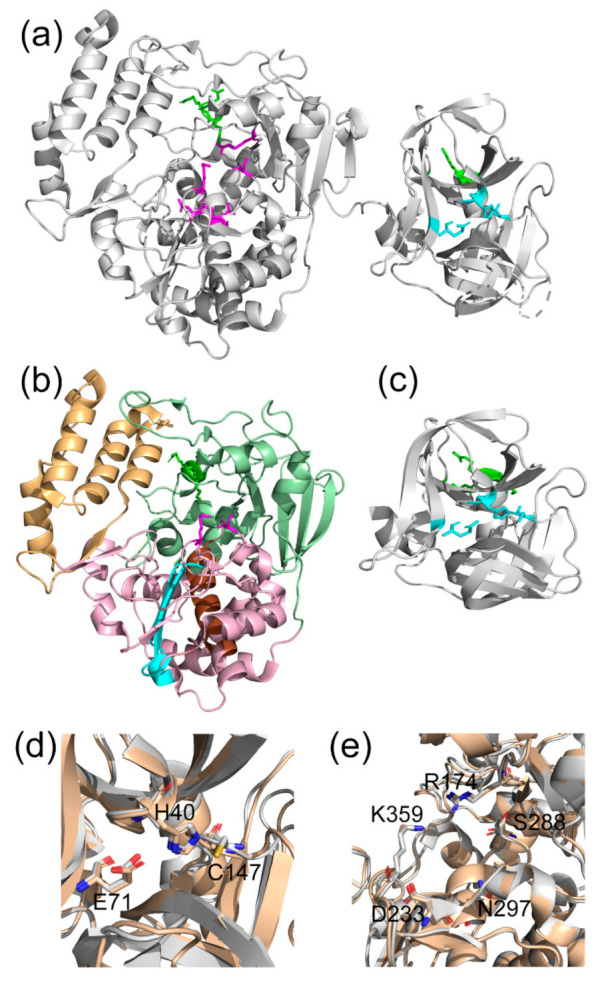
Structural comparison of poliovirus 3C, 3D, and 3CD proteins. (**a**) X-ray crystal structure of poliovirus 3CD (PDB: 2IJD). (**b**) X-ray crystal structure of poliovirus 3D RNA-dependent RNA polymerase (PDB: 1RA6). The subdomains of 3D are colored: palm – purple, fingers – green, thumb – orange. Motifs B and C in the palm subdomain are also colored, brown and cyan, respectively. (**c**) X-ray crystal structure of poliovirus 3C protease (PDB: 1L1N). The protease catalytic triad is shown in cyan, polymerase active site residues are shown in magenta, and RNA/PIP binding region is shown in green. (**d**) Active sites of 3C (gray) and 3C domain of 3CD (brown). (**e**) Active sites of 3D (gray) and 3D domain of 3CD (brown).

Poliovirus 3C, 3D and 3CD are all important proteins for the viral replication cycle. Here we will briefly discuss the structure and function of each. Structurally, 3C is cysteine protease with a chymotrypsin-like fold with the active site between two six-stranded β-barrels, with four helices (Figure 1). The catalytic triad consists of the general base His40, Glu71, and the nucleophilic Cys147, with Gly145, Gln146 and Cys147 contributing to the oxyanion hole [19]. The 3C protein also has an RNA binding site distant from the active site consisting mostly of residues 81–89 [20]. Overlapping with the RNA binding site is a phosphoinositide (PIP) binding site, important for lipid membrane interactions, which includes residues Lys12 and Arg13 in the N-terminal helix [21]. 3C can cleave most Gln/Gly junctions in the poliovirus polyprotein and interferes with cap-dependent translation, but only 3CD cleaves the capsid precursor P1 polyprotein [5,9,22]. While this finding indicates that the substrate specificity is different between 3C and 3CD, a structural explanation is lacking. Amino acid changes in the 3D domain distant from the 3C domain result in changes in substrate specificity [9,23], suggesting that the 3D domain has an allosteric effect on the protease active site that is not readily understood based on the X-ray crystal structures.

3D, the RNA-dependent RNA polymerase (RdRp), has the “cupped right hand” topology common to most nucleic acid polymerases [24], consisting of a fingers, palm, and thumb sub-domains (Figure 1). There are six conserved motifs, labeled A through F, within the palm and fingers domains. The active site consists of a three-stranded β-sheet with strands from motifs A and C and a long α-helix from motif B. Motifs A and C contain conserved aspartate residues, Asp233 and Asp328, which coordinate metal ions essential for catalysis. On motif B, Ser288 and Asn297 are important for recognition of the ribose sugar. Removing N-terminal residues of 3D or adding C-terminal residues of 3C to 3D decreases polymerase activity [25,26,27,28], suggesting that allosteric effects from the N terminus are important for polymerase function. While the structural basis of polymerase activation has been studied [29], differences in conformational dynamics in the context of 3CD may be important for understanding the lack of polymerase activity and the expanded functionality of 3CD compared to 3C and 3D. Given the structural similarity of 3D and 3CD, it has been proposed that molecular flexibility, rather than just a difference in structure, is an important determinant of polymerase activity [8,30,31].

Poliovirus 3CD is largely a structural composite of the 3C and 3D proteins, with a short linker connecting these domains (Figure 1). Unlike 3CD in some other RNA viruses (e.g., norovirus [32]), poliovirus 3CD does not function as a RdRp [30], although its 3D domain apparently modifies the functions of the 3C domain [9,10]. In addition to its proteolytic activity, 3CD is responsible for different interactions with RNA [14,15,16], induces formation of double-membrane vesicles [11,12,13] and likely interacts with PIP lipids in virus replication membranes [12]. Compared to the protease activity of 3C, 3CD cleaves the P1 capsid polyprotein and cleaves the other Gln-Gly junctions other than the P2/P3 junction more efficiently [9,10,22]. The different protease specificity between 3C and 3CD allows for temporal control over replication and translation via regulation of the relative amounts of each viral protein resulting from polyprotein cleavage [6,33,34].

The distinct and emergent functions of 3CD compared to the fully processed 3C and 3D proteins cannot be explained by structural considerations alone, given the nearly identical X-ray crystal structures of 3C/3D compared to 3CD (Figure 1). It is now appreciated that protein conformational dynamics play important roles in determining protein function. Specifically, conformational dynamics have been previously been shown to be important for protease activity and substrate specificity for many other systems [35,36,37,38,39] and the conformational dynamics of viral RdRps have likewise been shown to play critical roles in replication speed and fidelity [40,41,42,43,44]. Conformational dynamics and protein flexibility are also important in structure-based drug design [45,46,47,48,49]. Domain-domain motions within 3CD may also be functionally important; small angle X-ray scattering (SAXS) and molecular dynamics (MD) simulations have indicated that 3CD likely adopts multiple conformations in solution [18].

Here, we have used nuclear magnetic resonance (NMR) based methods to explore the conformational dynamic differences between 3C, 3D and 3CD as a potential explanation for their different functions. These experiments capture conformational dynamics across many timescales, including chemical exchange saturation transfer (CEST) [50] that monitors conformational exchange on the millisecond-to-second (ms-s) timescale, Carr-Purcell-Meiboom-Gill (CPMG) relaxation dispersion [51] that monitors conformational exchange on the microsecond-to-millisecond (µs-ms) timescale, and relaxation violated coherence transfer [52], which allows determination of conformational dynamics from the picosecond-to-nanosecond (ps-ns) timescale [53,54]. Conformational dynamics across these multiple timescales may be functionally important, with loop motion and side chain rotation occurring on the picosecond-to-nanosecond (ps-ns) timescale [55,56] and larger domain motions associated with ligand binding, allosteric regulation and catalytic turnover generally occurring on the µs-ms timescale [57,58]. Our studies reveal conformational dynamic differences between 3C/3D and 3CD in functionally relevant regions, suggesting that these dynamic differences may help to differentiate function between the polyprotein precursor and the fully processed proteins. Conformational dynamics within 3CD may also provide means of allosteric communication between the 3C and 3D domains, important for functional coordination of their activities.

## 2. Materials and Methods

### 2.1. Protein Expression and Isotopic Labeling

pSUMO plasmids with C-terminal hexahistidine tag expressing PV 3C, 3D, and 3CD were transformed into *Escherichia coli* BL21(DE3) pRARE cells. In 3D and in the 3D domain of 3CD, the following amino acid substitutions (relative to type 1 Mahoney strain) were present to prevent aggregation: L446D and R455D. In 3C and in the 3C domain of 3CD, the following amino acid substitutions were present to prevent aggregation in 3CD: E55A, D58A, E63A, and C147A to prevent intermolecular cleavage of 3CD [8]. Although not necessary to prevent aggregation in 3C, these mutations were present in 3C to make sure any differences in conformational dynamics were not due to these mutations. The aggregation-preventing mutations disrupt potential oligomerization interfaces on 3C and 3D and have been found to be necessary for achieving the high concentrations of 3CD necessary for NMR studies. Unless otherwise noted, reagents were purchased from VWR (Radnor, PA, USA).

After transforming cells with plasmids, cells were grown in 10 mL M9 minimal media (6.0 g/L Na_2_HPO_4_, 3.0 g/L KH_2_PO_4_, 0.5 g/L NaCl, 1.0 g/L NH_4_Cl, 2 g/L glucose, 1 mM MgSO_4_, 0.1 mM CaCl_2_, MEM vitamin mix (from Thermo Fisher, Bellefonte, PA, USA), trace metal mix (from Teknova, Hollister, CA, USA), 50 μg/mL kanamycin, 30 μg/mL chloramphenicol) for 16–20 h until optical density at 600 nm (OD_600_) was greater than 0.6. For ^15^N labeling, 50 mL M9 media were inoculated with 1 mL of the 10 mL culture and shaken overnight at 30 °C. Then, 20 mL were added to 1 L of M9 media (modified to contain 1 g/L ^15^N NH_4_Cl from Cambridge Isotope Laboratories, 12.0 g/L Na_2_HPO_4_, 6.0 g/L KH_2_PO_4_, 2 mM MgSO_4_). Protein production was initiated with 1 mM isopropyl-β-D-1-thiogalactopyranoside (IPTG) when OD_600_ was between 0.6–0.8 and the temperature was set to 25 °C. For perdeuterated Ileδ1-[^13^C,^1^H] labeling, 10–100 μL from the 10 mL M9 culture were plated onto M9 agar plates and incubated for 18–22 h at 37 °C. M9 minimal media (10 mL) with 50% D_2_O (D, 99% from Cambridge Isotope Laboratories, Andover, MA; USA) was inoculated with colonies from the M9 plate for 16–20 h until OD_600_ > 0.6, then 1 mL was transferred to 50 mL M9 minimal media with 100% D_2_O for 12–24 h until OD_600_ > 0.6. Cells (20 mL) were then added to 950 mL of 100% D_2_O M9 media. In the 100% D_2_O M9 media, glucose was replaced with deuterated glucose (1,2,3,4,5,6,6-D7, 97–98%, from Cambridge Isotope Laboratories, Andover, MA, USA) and the concentration of potassium phosphate buffer and magnesium sulfate were doubled [59]. Protein production was initiated with 1 mM IPTG when OD_600_ was between 0.6–0.8 and the temperature was set to 25 °C; 70 mg/L α-ketobutyric acid (Methyl-13C, 99% 3,3-D2, 98%; from Cambridge Isotope Laboratories, Andover, MA, USA) was added an hour before induction (around OD_600_ = 0.5). Cells were harvested after 16–20 h by centrifugation (3900× *g*, 30 min, 4 °C), rinsed with buffer (10 mM Tris, 1 mM EDTA pH 8), centrifuged again (3800× *g*, 15 min, 4 °C), decanted and the resulting pellets were weighed and frozen at −80 °C. All cultures were incubated at 37 °C with shaking at 200–250 rpm, unless otherwise specified. D_2_O (D, 99.9%) was from Cambridge Isotope Laboratories (Andover, MA, USA).

### 2.2. Protein Purification 

PV 3D and 3CD pellets were resuspended in 100 mM potassium phosphate pH 8.0, 10 mM β-mercaptoethanol, 120 uM ZnCl_2_, 20% glycerol, 1.4 μg/mL pepstatin A, 1 μg/mL leupeptin, 500 μM phenylmethanesulfonyl fluoride (PMSF). PV 3C pellets were resuspended in 20 mM HEPES pH 7.5, 50 mM NaCl, 1 mM EDTA, 5 mM β-mercaptoethanol, 5 mM imidazole, 1.4 μg/mL pepstatin A, 1 μg/mL leupeptin. The cells were then lysed, followed by polyethylenimine (PEI) precipitation and ammonium sulfate precipitation to 60% saturation as previously described [8]. The resulting pellet was resuspended and the proteins were purified by Nickel-Nitrilotriacetic Acid (Ni-NTA) affinity chromatography as previously described [20,60]. After cleavage of the SUMO tag by 1–2 μg ubiquitin-like-specific-protease (ULP-1), samples were dialyzed against 100 mM potassium phosphate, 100 mM sodium chloride, 60 μM zinc chloride, 5 mM β-mercaptoethanol, 20% glycerol. The samples were then concentrated using 30 kD molecular weight cutoff (MWCO; 3D and 3CD) or 10 kD MWCO (3C) Sartorius Vivaspin spin concentrators.

### 2.3. NMR Sample Preparation 

To prepare for methyl NMR spectroscopy, samples were buffer exchanged to 100 mM potassium phosphate, 100 mM sodium chloride, 60 μM zinc chloride, 5 mM β-mercaptoethanol, 20% d-8 glycerol (Cambridge Isotope Laboratories, Andover, MA, USA) in D_2_O using Zeba desalting columns (Thermo Scientific, Bellefonte, PA, USA). For ^15^N labeled samples, the buffer contained 90% H_2_O and 10% D_2_O. Samples were further concentrated by 0.5 mL Millipore Amicon Ultra Centrifugal Filters, 3K MWCO. The concentration was determined by measuring the absorbance at 280 nm (ε_3CD_ = 84690 M^−1^ cm^−1^, ε_3D_ = 75750 M^−1^ cm^−1^, ε_3C_ = 8960 M^−1^ cm^−1^). For determination of methyl axis order parameters, the concentration of all samples was 400 μM. All NMR experiments were carried out on 850 MHz Bruker Avance III or 600 MHz Bruker Avance NEO with 5 mm TCI single-axis gradient cryoprobes (Bruker, Billerica, MA, USA). Perdeuterated Ileδ1-[^13^C,^1^H] samples and uniformly labeled ^15^N 3C were prepared as described above. Data was processed using NMRpipe software [61] on NMRbox [62]. The ^1^H-^13^C heteronuclear multiple quantum coherence (HMQC) spectra are from the first plane of the triple quantum relaxation violated coherence transfer allowed experiment. For ^1^H-^13^C spectra, 64 points were used in the indirect dimension (t1) and 768 points were used in the direct dimension (t2).

### 2.4. δ1-13. CH_3_ Resonance Assignments for 3C, 3D, and 3CD

Resonance assignments for the δ1-^13^CH_3_ groups of 3C were determined using three-dimensional correlation experiments described in ref [63] using previously established backbone assignments [20]. Resonance assignments for 3D were determined by comparisons against a series of HMQC spectra of single Ile-to-Leu or Ile-to-Met variants in which assignments were made according to the missing resonances in the variants. Resonance assignments for 3CD were readily transferred over from the 3C and 3D spectra, as there were only small chemical shift differences and limited resonance overlap between 3C and 3D. Additional results of these experiments will be published elsewhere.

### 2.5. Determination of Methyl Axis Order Parameters 

Triple quantum relaxation violated coherence transfer experiments were performed at 600 MHz at 298 K and analyzed as previously described [64]. The allowed and forbidden experiments were interleaved. For 3C, delays of 1.5, 5, 3, 15, 8, 2, 10, 4, 6, 12.5, 2.5, 9, 5, 3.5, 7, 1.5 ms were used. For 3CD, delays of 1.7, 3.4, 1.2, 6, 0.4, 4.2, 0.8, 1.2, and 2.2 ms were used. For 3D, delays of 1.7, 3.8, 1.3, 10, 7, 0.4, 4.5, 0.9, 1.3, 2.5, and 5.4 ms were used.

The data were fit to Equation (1):

I_a_/I_b_ = ¾ (ηtanh((η^2^ + δ^2^)^1/2^ T))/((η^2^ + δ^2^)^1/2^ − δtanh((η^2^ + δ^2^)^1/2^T)) (1)

where I_a_/I_b_ is the ratio of intensities of the forbidden and allowed experiments, η is the intramethyl ^1^H-^1^H cross-correlated relaxation rate, δ is a parameter that accounts for relaxation due to external protons, and T is the relaxation delay period. η and δ for each residue were determined by nonlinear least squares fitting using the Trust Region Reflective algorithm. Uncertainty in fit parameters calculated by Monte Carlo bootstrapping resampling from a normal distribution using the standard deviation of repeating measurements was very small, so residual bootstrapping was used to determine uncertainty. 

Methyl axis order parameters (S^2^) were calculated according to Equation (2):

η = 9/10 (μ_0_/4π)^2^ (P_2_(cosθ_axis_,_HH_))^2^ (S^2^γ_H_^4^ℏ^2^τ_c_)/(r_HH_^6^) (2)

where τ_c_ is the rotational correlation time for the protein molecule, μ_0_ is the vacuum permittivity constant, γ_H_ is the gyromagnetic ratio for a proton spin, r_HH_ is the distance between methyl proton pairs, P_2_(x) is the second order Legendre polynomial 1/2(3x^2^−1), θ_axis,HH_ is the angle between the methyl 3-fold axis and a vector connecting a pair of methyl protons [64]. For 3C and 3D, calculations by HydroNMR predict that the rotational diffusion tensor is isotropic (D_∥_/D_⊥_ < 1.17 [65]), so we used a single isotropic τc for all residues. Calculation of order parameters with separate τ_c_ for each residue using an axially symmetric model results in a difference in order parameters of less than 3%. For 3CD, the rotational diffusion tensor calculated by HydroNMR [66] is approximately axially symmetric (D_∥_/D_⊥_ = 1.66), raising the possibility that the methyl 3-fold axis orientation relative to the principal axis of the diffusion tensor could affect the intramethyl cross- relaxation rate, with bond vectors aligned along the principal axis experiencing lower rotational correlation times than bond vectors aligned perpendicular [67]. However, there is good agreement between relative order parameters calculated using a single isotropic τ_c_ and relative order parameters calculated using different values of τ_c_ for each residue calculated from the axially symmetric rotational diffusion tensor [67,68,69], suggesting that the effect of anisotropic rotational diffusion on the interpretation of our results is small. Furthermore, because of the flexible linker, the domains are expected to tumble somewhat independently, leading to a more isotropic rotational diffusion for each domain than for the whole molecule tumbling together.

### 2.6. CPMG Relaxation Dispersion

^1^H-^13^C multiple quantum CPMG relaxation dispersion experiments were performed at 850 MHz at 295 K and 298 K, as previously described [70], with a total CPMG period of 20 ms and CPMG frequencies of 50, 1000, 500, 250, 800, 150, 600, 900, 200, 400, 100, 300, 400, 700, and 800 s^−1^. Error bars were calculated from spectral noise. R_ex_ was calculated by subtracting the value of R_2,eff_ at the highest ν_cpmg_ from R_2,eff_ at the lowest ν_cpmg_ for each residue. If both values of R_2,eff_ were within error due to noise, R_ex_ was considered to be 0. Curves for Ile151 in 3C and 3CD were fit using GUARDD in MATLAB [71].

### 2.7. H and ^13^C CEST

^1^H chemical exchange saturation transfer (CEST) experiments were performed at 850 MHz at 298 K, as previously described [72]. An exchange period of 500 ms was used, with saturation frequencies varied from −0.65 to 1.05 ppm for 3C and −0.89 to 1.33 ppm for 3D and 3CD, with a step size of 30 Hz. The B1 frequency was 30 Hz.

^13^C CEST experiments were performed at 850 MHz at 298 K on 3C, as previously described [73]. An exchange period of 40 ms was used, with saturation frequencies varied from 7.26 to 13.95 ppm with a step size of 25 Hz. The B1 frequency was 25 Hz.

## 3. Results

As the structural similarities between 3C and 3D with their corresponding domains in 3CD largely failed to explain functional differences, we sought to understand these differences by comparing conformational dynamics via NMR spectroscopy. Since our interest is primarily in comparing the conformational dynamics between the fully processed 3C and 3D proteins to their counterpart domains in the 3CD protein, we report first on NMR experiments with 3C and comparisons against the 3C domain of 3CD, before reporting on the NMR experiments of 3D and comparisons against the 3D domain of 3CD. At the end of this section, we then compare the rotational correlation times of 3C and 3D by themselves and in the context of 3CD to learn about the degree of independence of the domains in 3CD. To avoid confusion, residues belonging to the 3C and 3D proteins will be identified according to 3C and 3D superscripts, respectively. We will also refer to residues in the 3C domain of 3CD with a superscript 3C*D and residues in the 3D domain of 3CD with a superscript 3CD*. In this way, we can keep the numbering convention for 3D while referring to the corresponding domain in 3CD.

### 3.1. Ile Methyl Resonance Assignments for 3C

**Figure 2:**
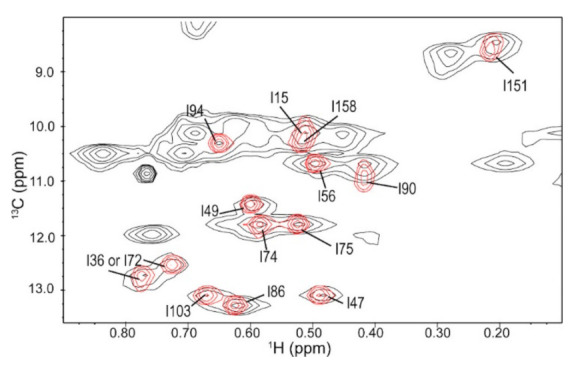
^1^H-^13^C heteronclear multiple quantum coherence (HMQC) Ile methyl NMR spectrum of 3C (red) and 3CD (black).

^1^H-^13^C methyl HMQC NMR spectra were collected for poliovirus 3C labeled with ^13^C at the delta-1 carbon of all Ile residues (Figure 2). Conventionally, ^15^N in the amide backbone is used in two-dimensional protein NMR, but there is too much spectral overlap in the spectrum of 3CD. By labeling the isoleucine methyl groups, we were able to avoid this spectral overlap. Furthermore, methyl groups are especially sensitive probes for large proteins such as 3CD due to the methyl TROSY effect [74]. Isoleucine was chosen as the NMR probe because these residues are well-dispersed throughout the protein structure and are well resolved in the spectrum, except for Ile15^3C^ and Ile158^3C^. The methyl δ1-[^13^C,^1^H] Ile resonances were assigned using 3D correlation experiments as described in ref. [63] based on backbone resonance assignments reported in ref. [20]. All residues appear in the spectrum as single resonances and do not appear to be noticeably exchange-broadened.

### 3.2. Conformational Dynamics of 3C on the Micro-to-Millisecond Timescale

**Figure 3:**
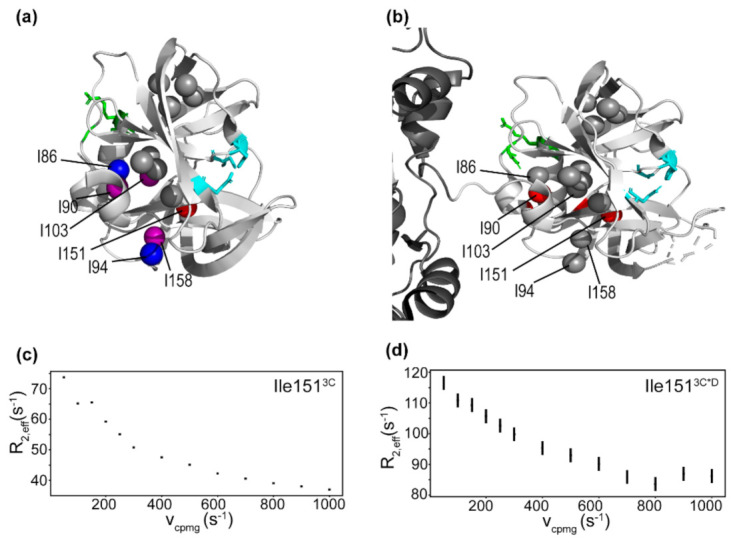
Conformational exchange processes in 3C and the 3C domain of 3CD on the µs-ms timescale. (**a**) R_ex_, the contribution to relaxation from conformational exchange, from multiple quantum ^1^H-^13^C CPMG (Carr-Purcell-Meiboom-Gill) relaxation dispersion for 3C is plotted as colored spheres (gray: no detectable exchange, blue < 2–4 s^−1^, purple: 4–10 s^−1^, red > 10 s^−1^). The protease active site is shown in cyan and the RNA binding site is shown in green. (**b**) Multiple quantum ^1^H-^13^CHHHH CPMG relaxation dispersion curve for Ile151 in 3C. The effective relaxation rate R_2eff_ is plotted against the CPMG pulsing frequency. (**c**) R_ex_ from multiple quantum ^1^H-^13^C CPMG relaxation dispersion for 3CD is plotted as colored spheres (gray: no detectable exchange, red > 10 s^−1^). (**d**) Multiple quantum ^1^H-^13^CHHHH CPMG relaxation dispersion curve for Ile151 in the 3C domain of 3CD.

Multiple-quantum ^1^H-^13^C CPMG relaxation dispersion experiments were performed on 3C to provide insight into conformational exchange events occurring on the µs-ms timescale. In short, CPMG relaxation dispersion measures the difference in intensity of resonances as nuclear spin relaxation due to conformational exchange is refocused by a train of rapid pulses, and can determine exchange parameters for residues exchanging on a timescale comparable to the frequency of the refocusing pulses [51,75]. Residues with detectable conformational exchange include Ile86^3C^, Ile90^3C^ and Ile94^3C^ on the RNA binding loop [19,20], Ile103^3C^ that contacts the PIP-binding N-terminal helix [21], Ile151^3C^ on the same loop as the active site nucleophile Cys147, and Ile158^3C^/Ile15^3C^ (Figure 3 and Figure S1). Ile151^3C^ has a particularly large contribution to relaxation from exchange, indicating that it is probably exchanging between states with large differences in chemical shift. One possible explanation for the large contribution to relaxation from exchange is that the δ1 methyl of Ile151 contacts the hydroxyl of Tyr138, which is on a substrate-binding loop. The μs-ms timescale dynamics observed here are consistent with previous backbone ^15^Ν CPMG relaxation dispersion studies in which it was found that residues in the active site were dynamic on the μs-ms timescale [76]. It is possible that at least some of the apparent dynamics in the active site are due to protonation/deprotonation of His40 [77], although Ile151 is likely too far away to be directly affected. Given residues in and around the active site are dynamic on a catalytically relevant timescale, these dynamics may be important for the selection of a protease active conformation and/or substrate specificity.

### 3.3. Conformational Dynamics of 3C on the Millisecond-Second Timescale

**Figure 4:**
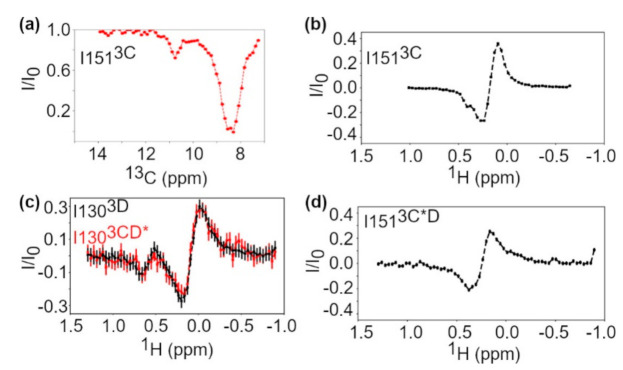
Chemical exchange saturation transfer (CEST) profiles for selected residues in 3C, 3D, and 3CD. Error bars were calculated from spectral noise. (**a**) ^13^C CEST profile for Ile151 in 3C. The decrease in intensity at 8.5 ppm is because the ^13^C chemical shift for Ile151 is at 8.5 ppm. The decrease around 11 ppm is due to conformational exchange. (**b**) ^1^H CEST profile for Ile151 in 3C. For the ^1^H CEST experiments used here, the measured intensity is only non-zero when the observed resonance or a state it is exchanging to has ^1^H chemical shift around the saturating frequency. (**c**) ^1^H CEST profile of Ile130 in 3D (black) and 3CD (red, 3D numbering). (**d**) ^1^H CEST profile for Ile151 in 3CD.

CEST experiments were also performed, which provide insight into conformational exchange on the ms-s timescale. Briefly, CEST measures the decrease in resonance intensity when a weak transverse magnetic field is applied at the resonance frequency. When a residue is exchanging to a state, unobservable in the standard correlation spectrum, whose resonance frequency is saturated by the transverse field, the intensity of the observable resonance decreases as the chemical shift of the normally invisible exchanging state is saturated [50,78]. The CEST experiments indicate that Ile151^3C^ is undergoing conformational exchange, in which the minor state has substantial ^1^H and ^13^C chemical shift differences with the major state (Figure 4a,b). This result is consistent with conformational exchange as detected by the relaxation dispersion experiment (Figure 3). Other active site residues do not appear to be exchanging on this timescale (Figures S2 and S3), but there are limited probes in the active site and the chemical shift difference for the Ile residues may be too small to observe the minor state.

### 3.4. Conformational Dynamics of 3C on the Picosecond-Nanosecond Timescale

**Figure 5:**
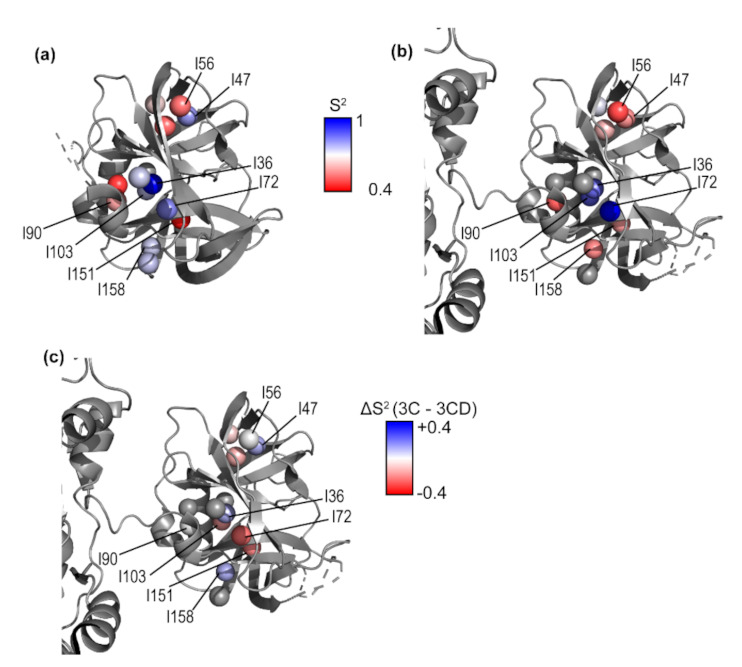
Methyl axis order parameters for 3C and the 3C domain in 3CD. Methyl axis order parameters are plotted as colored spheres ranging from 0.4–1 (red to blue) as indicated for (**a**) 3C and (**b**) 3C domain of 3CD. (**c**) Differences in methyl axis order parameters between 3C and 3CD (ΔS^2^ = S^2^ (3C) − S^2^ (3CD)) are plotted as colored spheres, ranging from −0.4 to +0.4 (red to blue).

While slower conformational dynamics include potentially functionally relevant larger movements such as domain motions, faster motions such as loop motions on the ps-ns timescale may also be important for substrate binding or allostery [55,56]. Methyl axis order parameters (S^2^), which report on ps-ns timescale dynamics [79], were determined by triple quantum relaxation violated coherence transfer NMR experiments [64,80] (Figure 5 and Figure S4). Using a rotational correlation time (τ_c_) of 24.8 ns so that the highest order parameter was 1, the average value of the order parameters calculated for 3C was 0.69. This value for τ_c_ agrees very well with the value calculated by HydroNMR (25.1 ns). Order parameters calculated using the τ_c_ determined by TRACT NMR are given in parentheses (see discussion Figure S5). Ile36^3C^ and Ile72^3C^ have the highest methyl axis order parameters, 1.0 and 0.83 (0.86 and 0.72). The side chains of Ile36^3C^ and Ile72^3C^ are contacting via hydrophobic interactions, and Ile72^3C^ is adjacent to the active site residue Glu71, which is part of the catalytic triad. Ile151^3C^ has the lowest methyl axis order parameter in 3C, 0.46 (0.39), indicating that it is the most dynamic on the ps-ns timescale.

### 3.5. Conformational Dynamics of 3CD: 3C Domain

One of our major goals was to compare and contrast internal motions between 3CD and the corresponding 3C and 3D proteins. As such, we performed similar experiments (i.e., multiple quantum relaxation dispersion, CEST and methyl axis order parameters) for 3CD (Figures S6–S8). Here, we report only on the 3C domain, and leave the analysis of the 3D domain until after reporting on the 3D conformational dynamics in the next sections.

Conformational exchange processes on the μs-s timescales were quite similar between the 3C protein and the 3C domain of 3CD (Figure 3 and Figure 4). In particular, residues with detectable exchange in the 3C domain included Ile90^3C*D^ in the RNA-binding loop and Ile151^3C*D^ in the protease active site. Ile90^3C*D^ may be associated with a larger R_ex_ value in 3CD suggesting changes in kinetics and/or thermodynamics associated with conformational exchange, and/or a change in the exchanging conformation. It is notable that there was also a small chemical shift difference for Ile90 between 3C and 3CD (Figure 2). Ile86 and Ile94, which are exchanging in 3C, may not be exchanging in 3CD (Figure 3), although the relaxation dispersion curves are generally noisier for 3CD. The CEST experiments also indicated that Ile151 may be exchanging in 3C but not in 3CD, which again may be suggestive of a difference in timescale for Ile151 exchange between 3C and 3CD (Figure 4b,d).

For analyzing methyl axis order parameters in 3CD, the rotational correlation time (τ_c_) was determined by assuming the highest order parameter was 1 because residues in the 3C and 3D domains could not be analyzed for τ_c_ separately due to ^1^H-^15^N resonance overlap. More specifically, measurement of the rotational correlation time by the TRACT NMR experiment resulted in a value lower than used here because without separation of the resonances in the ^1^H and ^15^N dimensions, slower-relaxing resonances overpower the faster-relaxing resonances that are needed to accurately determine τ_c_ [81]. 

The highest methyl axis order parameters in the 3C domain of 3CD were for Ile36^3C*D^, Ile72^3C*D^, and Ile103^3C*D^. Ile36^3C*D^ and Ile72^3C*D^ are near the active site residue Glu71 and Ile103 is near the RNA binding site. The residues with lowest order parameters were Ile56^3C*D^ and Ile90^3C*D^. Ile56^3C*D^ is next to Val54 which when mutated reduces the processing of 3CD [82]. These results largely mirrored the results for the 3C protein (Figure 5), although some residues (e.g., Ile47^3C*D^, Ile151^3C*D^) appeared to have higher order parameters, while other residues (e.g., Ile72^3C*D^) had lower order parameters for 3C compared to 3CD (Figure 5c). It should be noted that the order parameter values we calculated depend on the τ_c_ values, which we can only estimate for 3CD but the analysis presented here is expected to be valid for a wide range of τ_c_ values. That is, these results indicate that there are at least some small relative changes in the ps-ns timescale dynamics in the 3C domain of 3CD compared to the isolated 3C protein.

### 3.6. Ile Methyl Resonance Assignments for 3D

**Figure 6:**
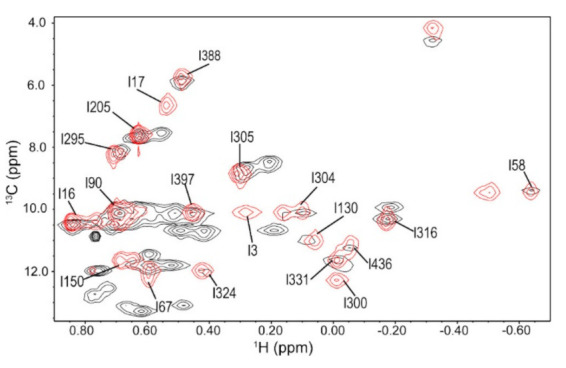
^1^H-^13^C-HMQC Ile methyl NMR spectra of 3D (red) and 3CD (black). 3D numbering is used here for both 3D and the 3D domain in 3CD; to find the corresponding number in 3CD, the reader would need to add 183.

We used a mutagenesis approach to assign the δ1-^13^CH_3_ Ile resonances for 3D. Briefly, we compared HMQC spectra of 3D against a series of Ile-to-Leu and Ile-to-Met variants, so that missing resonances in the variants were assigned to the corresponding substituted Ile residue. In most cases, these assignments were straightforward, although some additional chemical shift changes were noted. We will report on these mutagenesis studies and their implications for allostery in 3D in a future manuscript, as a comprehensive analysis of these results would distract from, and are not necessarily pertinent to, the current analysis. Germane for the current manuscript, these experiments allowed us to assign most of the δ1-^13^CH_3_ Ile resonances for 3D (Figure 6).

### 3.7. Conformational Dynamics of 3D on the Microsecond to Second Timescale

**Figure 7:**
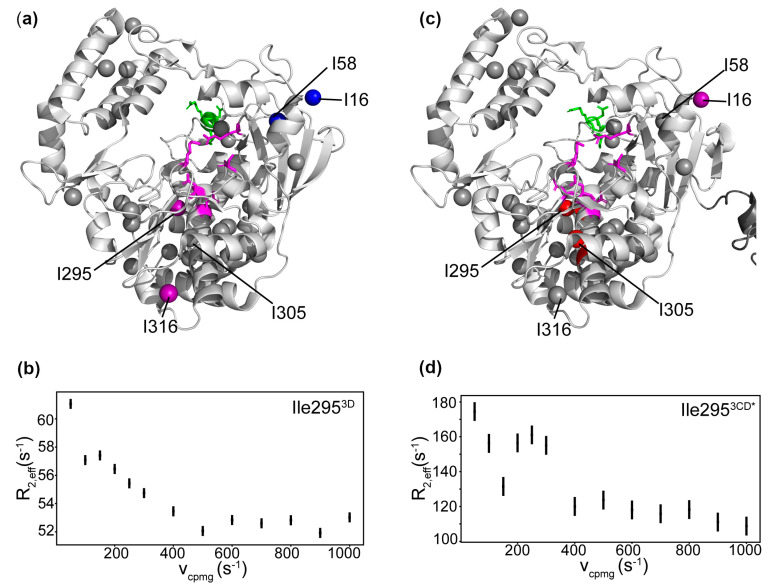
Conformational exchange processes in 3D and the 3D domain of 3CD on the μs-ms timescale. (**a**) R_ex_, the contribution to transverse relaxation from conformational exchange, from multiple quantum ^1^H-^13^C CPMG relaxation dispersion for 3D plotted as colored spheres (gray: no detectable exchange, blue < 2–3 s^−1^, purple: 3–10 s^−1^). (**b**) Multiple quantum ^1^H-^13^CHHHH CPMG relaxation dispersion curve for Ile295 in 3D. (**c**) R_ex_ from multiple quantum ^1^H-^13^C CPMG relaxation dispersion for 3CD plotted as colored spheres (gray: no detectable exchange, purple: 3–10 s^−1^, red > 10 s^−1^). (**d**) Multiple quantum ^1^H-^13^CHHHH CPMG relaxation dispersion curve for Ile295 in the 3D domain of 3CD. 3D numbering is used here for both 3D and the 3D domain in 3CD; to find the corresponding number in 3CD, the reader would need to add 183.

We collected multiple quantum relaxation dispersion and CEST experiments to likewise identify μs-s conformational exchange events in the 3D protein (Figures S9 and S10). Based on the relaxation dispersion experiments, Ile295^3D^ and Ile316^3D^, located in the palm subdomain, had the largest contribution to relaxation from exchange (Figure 7). Ile16^3D^ and Ile58^3D^, located in the fingers subdomain, appeared to have small but observable contributions to relaxation from exchange. Ile295^3D^ is located on motif B, and Ile316^3D^ is on the loop between motifs B and C. ^1^H CEST revealed a minor conformational state for Ile130^3D^ (Figure 4c), which is located in a potential PIP binding site [83]. Conformational exchange in the PIP binding site may be important for the conformational selection of a more binding-competent conformation.

### 3.8. Conformational Dynamics of 3D on the Picosecond to Nanosecond Timescale

**Figure 8:**
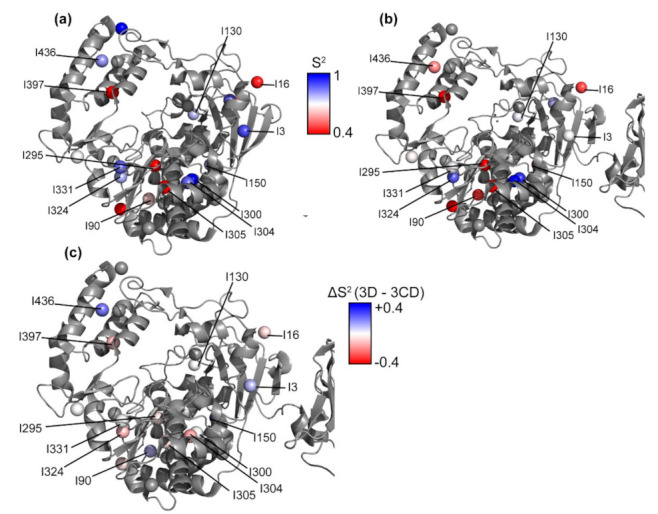
Methyl axis order parameters for 3D and the 3D domain in 3CD. Methyl axis order parameters are plotted as colored spheres ranging from 0.4–1 (red to blue) as indicated for (**a**) 3D and (**b**) 3D domain of 3CD. (**c**) Differences in methyl axis order parameters between 3D and 3CD (ΔS^2^ = S^2^ (3D) − S^2^ (3CD)) are plotted as colored spheres, ranging from −0.4 to +0.4 (red to blue). 3D numbering is used here for both 3D and the 3D domain in 3CD; to find the corresponding number in 3CD, the reader would need to add 183.

The ps-ns timescale dynamics were also investigated in 3D (Figure 8 and Figure S11). Using a rotational correlation time (τc) of 81 ns so that the highest order parameter was 1, the average value of the order parameters calculated for 3D was 0.64. The τ_c_ value was chosen this way because spectral overlap in the ^1^H-^15^N correlation spectrum would likely result in an underestimation of τc. The residues with highest order parameters were Ile300^3D^, Ile3^3D^, and Ile331^3D^ with values of 1.00, 0.90, and 0.88, respectively. Ile300^3D^ is in the palm domain in motif B, Ile3^3D^ is at the N-terminal loop of 3D, and Ile331^3D^ is in the palm domain in motif C. These residues have been proposed to stabilize the active polymerase conformation via a hydrogen-bonding network [29,60,84]. The residues with lowest order parameters were Ile205^3D^, Ile295^3D^, and Ile305^3D^ in the palm domain, and Ile397^3D^ in the thumb domain. Ile295^3D^ and Ile305^3D^ are located in motif B.

### 3.9. Conformational Dynamics of 3CD: 3D Domain

Here, we have used 3D numbering in the analysis of the 3D domain of 3CD, as the 3D RdRp is more commonly studied than the 3CD precursor protein. The reader would need to add 183 to the 3D numbering to get the corresponding residues in 3CD. In the 3D domain of 3CD, Ile16^3CD*^, lle295^3CD*^, and Ile305^3CD*^ were dynamic on the μs-ms timescale according to the multiple quantum relaxation dispersion experiments, similar to what was observed for 3D (Figure 8 and Figure S12). The ^1^H CEST experiment also revealed slower conformational dynamics in Ile130^3CD*^ in the 3D domain (Figure 4c and Figure S13). Unfortunately, many of the CEST profiles were noisy and difficult to interpret, likely due to the higher molecular weight of 3CD. 

The residues with highest methyl axis order parameters were all in the palm domain: Ile300^3CD*^, Ile304^3CD*^, and Ile324^3CD*^; Ile300^3CD*^ and Ile304^3CD*^ are in motif B, and Ile324^3CD*^ is in motif C. The residues with lowest order parameters were Ile295^3CD*^ and Ile305^3CD*^, also in the palm domain in motif B (Figure 8b and Figure S8). These results were quite similar to those for 3D, with some notable exceptions in which residues were associated with higher (e.g., Ile3, Ile90, Ile436) or lower (e.g., Ile304, Ile324) methyl axis order parameters for 3D compared to the 3D domain of 3CD. Again, it should be kept in mind that the methyl axis order parameters depend on the τ_c_ values, which were estimated for both 3D and 3CD. Nonetheless, these comparisons indicate that there are relative conformational dynamics differences within 3CD compared to 3D.

### 3.10. Domain Independence in 3CD

Having determined methyl axis order parameters for 3CD, we can gain some insight into the independence of the 3C and 3D domains in the context of 3CD by comparing the rotational correlation time (τ_c_) of the independent domains to the domains in the context of 3CD. The τ_c_ value can be thought of as the average time it takes for a molecule to rotate 1 radian. If the 3C and 3D domains in 3CD tumble independently and do not interact, τ_c_ for each domain would be less than τ_c_ for the whole protein considered as a rigid body, with τ_c_ for each domain roughly proportional to its molecular weight [85]. On the other hand, if their rotation was completely coupled, the τ_c_ values would be identical and larger than the τ_c_ of the individual domains [86,87]. As the magnitude of the methyl axis order parameters scale with the chosen value of τ_c_, we will focus on the value of the intramethyl ^1^H-^1^H cross-correlated relaxation rates, η (see Figures S4, S8 and S11), which is independent of any assumptions about τ_c_. 

**Figure 9:**
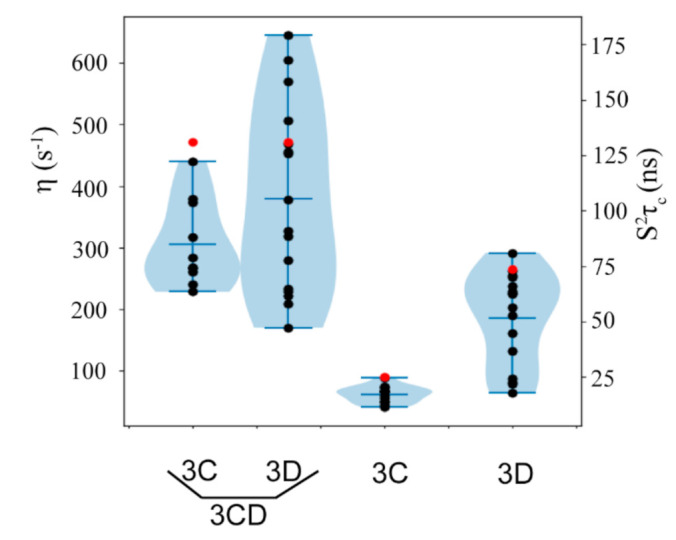
Distribution of intramethyl ^1^H-^1^H dipolar cross-correlated relaxation rates (η) and the equivalent S^2^τ_c_ values determined by triple quantum relaxation violated coherence transfer NMR experiments for the 3C and 3D domains independently and in 3CD. Values of τ_c_ calculated from HydroNMR are shown in red. Mean ± standard deviation of η: 3C: 62 ± 14 s^−1^, 3D: 186 ± 75 s^−1^, 3C in 3CD: 306 ± 70 s^−1^, 3D in 3CD: 379 ± 153 s^−1^.

For the 3C and 3D domains in the context of 3CD, the distribution of η values were more similar than when the two domains were not connected (Figure 9), although the 3D domain has higher values of η overall. The overlap of the η distribution suggests that the 3C and 3D domains in 3CD have somewhat similar τ_c_. Due to the linker, the domains are not expected to be completely independent, but the τ_c_ of 3CD is much larger than expected for non-interacting domains [85]. The larger τ_c_ in 3CD may be due to increased aggregation [88], inter-protein electrostatics [89], slow interdomain motions [90], or interdomain contacts [86,87]. That the 3D domain in 3CD undergoes a larger degree of nanosecond timescale motion than the 3C domain, and so would have a larger τ_c_ in 3CD, is supported by MD simulations [18]. While interdomain contacts do not fully explain why the τ_c_ in 3CD is so much larger than predicted by hydrodynamic calculations (160–180 ns vs. 130 ns), they could contribute to each domain having a larger τ_c_ by limiting the rotational diffusion, which would result in a longer rotational correlation time. Interdomain contacts, i.e., 3CD adopting a more compact conformation in solution than in the X-ray crystal structure (Figure 1), have been proposed based on SAXS and MD simulations [18].

## 4. Discussion

While poliovirus 3CD appears to be nearly structurally identical to its isolated domains (Figure 1), it is functionally different, enabling the virus to expand the functionality of its limited genome. 3CD has different protease specificity from 3C [9,10], and unlike 3D it does not function as a polymerase [30]. As these functional differences are not explained by existing structural information, it has been proposed that differences in conformational dynamics between 3CD and 3C/3D may be responsible for the different functionality [18]. Here we used NMR spectroscopy to compare residue-specific conformational dynamics between 3CD and its processed products in order to gain insight into the role of conformational dynamics in functional differences between the proteins. 

There is an important caveat when discussing the methyl axis order parameters (S^2^), especially in comparisons between 3CD and 3C/3D. To calculate order parameters from intramethyl cross-correlated relaxation rates (η), we used the simplest version of the Lipari-Szabo model-free formalism [52,79], where η depends on two parameters: S^2^ and τ_c_. Any comparison of order parameters between different proteins depends on the chosen values of τ_c_. When discussing order parameters, we are comparing the distribution of order parameters within the protein rather than absolute values of S^2^ because the true value of τ_c_ is not known for 3CD due to its large molecular weight and separate domains of 3CD making traditional methods for determining τ_c_ unavailable. To assess the accuracy of our use of the highest order parameter to determine τ_c_, we used the TRACT NMR experiment [81] to determine τ_c_ for 3C (see Supplementary Materials). The resulting value of 28.7 ± 1.6 ns is close to the value of 24.8 ns determined by assuming the highest order parameter is 1, and the differences in order parameters identified here are fairly large, so the qualitative analysis of differences in order parameters presented in the following discussion is expected to be valid.

### 4.1. Comparison of 3C and 3CD Conformational Dynamics

Multiple quantum ^1^H-^13^C CPMG relaxation dispersion NMR experiments were performed on 3C and 3CD to determine conformational dynamics on the μs-ms timescale. In both 3C and 3CD, Ile151^3C^, which is on the same loop as the active site nucleophile Cys147, appears to undergo conformational exchange on the μs-ms timescale (Figure 3 and Figure 4). Residues around Cys147 in 3C have previously been shown to undergo conformational exchange by ^15^N R_2_ relaxation dispersion experiments [76]. The conformational exchange kinetics may be different between 3C and 3CD, considering that the ^1^H CEST experiments were unable to identify a minor conformation for Ile151 (Figure 4), although it is noted that these experiments become more challenging to interpret with larger proteins. There also appeared to be differences for the ps-ns timescale dynamics around the active site. Ile151^3C^ has a higher apparent methyl axis order parameter in 3CD compared to 3C (Figure 5). Ile47^3C^, connected by a loop to the catalytic triad base His40, is less ordered in 3CD compared to 3C. The dynamic differences near the active site may be related to the different substrate specificity between 3CD and 3C, as has been proposed for other proteases [35,36,38,39]. 

There are also differences in conformational dynamics between the 3C protein and the 3C domain in 3CD outside of the protease active site. For example, there was a small upfield shift for Ile90^3C^ in the ^13^C dimension in 3CD relative to 3C (Figure 2). Ile90^3C*D^ is located near the linker between the 3C and 3D domains, and so its magnetic environment may be affected by the presence of the 3D domain. This residue also exchanges in both 3C and 3CD, although the kinetics, thermodynamics and/or exchanging conformation may be different (Figure 3). As the 3C and 3D domains do not interact in this region according to the X-ray crystal structure, these results may suggest that there is a difference in the average solution conformation for 3CD near the linker compared to the X-ray crystal structure.

While Ile158^3C^ (or Ile15^3C^; which have been difficult to resolve, although they are close in three-dimensional space) are exchanging in 3C, they do not appear to be exchanging in 3CD. Ile15^3C^ is adjacent to the PIP binding site containing Lys12 and Arg13. Ile94^3C*D^ and Ile103^3C*D^ also do not appear to undergo detectable exchange, although the resonances are of low intensity and the relaxation dispersion profiles were noisy, likely due to the slow tumbling time, and hence elevated transverse relaxation rate, of 3CD. Ile94^3C^ is adjacent to the RNA and lipid binding site in 3C [20,21,91]. The presence of the linked 3D domain may result in conformational dynamic differences important for the RNA binding differences between 3C and 3CD [14,15,16]. 

### 4.2. Comparison of 3D and 3CD Conformational Dynamics

Ile3^3D^, Ile300^3D^ and Ile331^3D^, which are in the palm domain in between the N terminus of 3D and the active site, have been proposed to stiffen the structure of the polymerase via a hydrogen bonding network [29,84]. These residues all have higher order parameters in 3D than in 3CD, confirming that the conformational dynamics are reduced in 3D compared to 3CD. The only residues that were found to be much more ordered in 3CD compared to 3D are Ile304^3D^ and Ile324^3D^, located in the palm domain. Ile324^3D^ is on motif C, which contains the metal-coordinating Asp328. Ile305^3D^ and Ile316^3D^, in the palm domain on motif B, are distant from the N terminus and 3C domain and appear to be in a very similar chemical environment in the x-ray crystal structures. However, they have different chemical shifts between 3D and 3CD. Ile305^3D^ is on the same α-helix as Asn297, which contacts the 2′ OH of the incoming NTP [92]. Ile300^3D^, also on this helix, does not appear in the 3CD spectrum indicating that it is likely broadened by conformational exchange. Ile295^3D^, also on motif B, has a much higher R_ex_ in 3CD than in 3D (Figure 7). This difference indicates that in 3CD, Ile295^3CD*^ is likely exchanging to a state with a larger difference in chemical shift and/or a higher relative population. Alternatively, Ile295^3D^ may be exchanging too fast for the contribution to relaxation from conformational exchange to be refocused as effectively by the CPMG pulse train. If the larger R_ex_ in 3CD is due to a higher excited state population, this would be consistent with the idea that 3CD adopts additional conformations in solution relative to its isolated domains [18]. Ile90^3D^ is less ordered in 3CD than in 3D; this residue contacts Phe258, which has been shown to be necessary for recruitment of guanine nucleotide exchange factors required for membrane reorganization in 3CD but not 3D and 3C separately [11]. 

Ile17^3D^ and Ile58^3D^, located near the N terminus of 3D in the fingers domain, have chemical shift differences between 3D and 3CD (Figure 6). These differences may be because the N terminus of 3D is tucked into the fingers domain whereas in 3CD it forms the linker between 3C and 3D. Residues Tyr118 and Tyr148 in the fingers domain have been shown to be important for initiation of RNA synthesis [93]. Ile130^3D^ and Ile150^3D^, near these two residues, also have chemical shift differences between 3D and 3CD (Figure 6) and both have higher order parameters in 3D than in 3CD (Figure 8c). These differences in structure and/or conformational dynamics may contribute to the lack of polymerase functionality in 3CD. ^1^H CEST experiments revealed that Ile130^3D^ is exchanging with a conformation that has a large difference in ^1^H chemical shift on the slow timescale in both 3D and 3CD. Insertion of four amino acids after Asp146, which is distant from the 3C domain, has been shown to decrease proteolytic processing of P1 by 3CD [23]. Ile150^3D^, which is on the same loop as Asp146, has a much higher order parameter in 3D than in 3CD, so the conformational dynamics observed for Ile150^3CD*^ may be important for allosteric communication with the 3C domain.

## 5. Conclusions

We identified differences in conformational dynamics on the picosecond to second timescales between the poliovirus 3C, 3D and 3CD proteins via NMR spectroscopy that may be important for understanding the differences in protease specificity, RNA and lipid interactions and polymerase activity. Differences in conformational dynamics near the protease active site and RNA binding site in 3C may be important for differentiating functions between the 3C and 3CD proteases. Similarly, enhanced flexibility of the 3D domain of 3CD may help to explain its lack of polymerase activity. Addition of protease substrates, RNA and/or PIP lipids may further accentuate these dynamic differences. Our results indicate that allosteric coordination between the 3C and 3D domains of 3CD likely operates through changes in these conformational dynamics. 

## Data Availability

The data presented in this study are reported in the current manuscript or Supplementary Materials.

## Supplementary Materials

The following are available online at https://www.mdpi.com/1999-4915/13/3/442/s1, Supplementary Methods: Validation of rotational correlation time τ_c_ by TRACT, Figure S1: Multiple quantum ^1^H-^13^C CPMG relaxation dispersion plots for δ1-methyl Ile groups in the 3C protein, Figure S2: ^1^H CEST profiles for δ1-methyl Ile groups in the 3C protein, Figure S3: ^13^C CEST profiles for δ1-methyl Ile groups in the 3C protein, Figure S4: Nonlinear fits for the intramethyl ^1^H-^1^H cross-correlated relaxation (eta), including the correction for dipolar interactions with external protons (delta) for δ1-methyl Ile groups in the 3C protein, Figure S5: Fitted ^1^H-^15^N TRACT results for determination of τ_c_ for 3C, Figure S6: Multiple quantum ^1^H-^13^C CPMG relaxation dispersion plots for δ1-methyl Ile groups in the 3C domain of the 3CD protein (i.e., 3C^*^D), Figure S7: ^1^H CEST profiles for δ1-methyl Ile groups in the 3C domain of the 3CD protein (i.e., 3C^*^D), Figure S8: Nonlinear fits for the intramethyl ^1^H-^1^H cross-correlated relaxation (eta), including the correction for dipolar interactions with external protons (delta) for δ1-methyl Ile groups in the 3CD protein, Figure S9: Multiple quantum ^1^H-^13^C CPMG relaxation dispersion plots for δ1-methyl Ile groups in 3D protein, Figure S10: ^1^H CEST profiles for δ1-methyl Ile groups in the 3D protein, Figure S11: Nonlinear fits for the intramethyl ^1^H-^1^H cross-correlated relaxation (eta), including the correction for dipolar interactions with external protons (delta) for δ1-methyl Ile groups in the 3D protein, Figure S12: Multiple quantum ^1^H-^13^C CPMG relaxation dispersion plots for δ1-methyl Ile groups in the 3D domain of the 3CD protein (i.e., 3CD^*^), Figure S13: ^1^H CEST profiles for δ1-methyl Ile groups in the 3D domain of the 3CD protein (i.e., 3CD^*^).
