# Supplementary material for: The Picornavirus Precursor 3CD Has Different Conformational Dynamics Compared to 3Cpro and 3Dpol in Functionally Relevant Regions
Source: Viruses. 2021 Mar 9;13(3):442. doi: 10.3390/v13030442 (PMC8001691; doi:10.3390/v13030442)
Supplement: Supplementary file 1 [file viruses-13-00442-s001.pdf]

Supplementary Materials

The picornavirus precursor 3CD has different conformational dynamics compared to $3C^{pro}$ and $3D^{pol}$ in functionally-relevant regions

Dennis S. Winston and David D. Boehr*

The Pennsylvania State University, University Park, PA, USA, 16802

*Corresponding Author: ddb12@psu.edu

Supplementary Methods

Validation of rotational correlation time τc by TRACT

1D [^{15}N-^{1}H]-TRACT experiments were performed as previously described [1] at 850 MHz at 298 K, with delay periods for determining Rα of 10, 40, 80, 20, 60, 100, 50, 70, and 10 ms and delay periods for determining Rβ of 0.02, 6, 2.8,8.4, 0.4, 1.6, 0.8, 10, 4.4, 0.2, 7.4, 2, 3.6, 5.2, 1.2, 20, 1.5, and 0.02 ms. For each delay, the resulting 1D spectra were integrated from 8-10 ppm. The resulting curves of integral versus delay period were fit for single exponential decay rates by nonlinear least squares with uncertainty determined by residual bootstrapping (Figure S5). Equations 1 and 2 were used to calculate τc [1]:

$$R_\beta - R_\alpha = 1/6\ (\mu_0/4\pi)\ (\hbar\gamma_N^2\gamma_H\ B_0\Delta\delta_N)/(r_{NH}^2)\ (3\cos^2\theta-1)(4J(0)+3J(\omega_N)) \quad (1)$$

$$J(w) = 2/5\ \tau_c/(1+w^2\tau_c^2) \quad (2)$$

where Rα and Rβ are the decay rates for the α and β spin states, μ_0 is the vacuum permittivity constant, γ_N and γ_H are the gyromagnetic ratios for ^{15}N and ^{1}H respectively, B_0 is the strength of the external magnetic field, $\Delta\delta_N$ is the difference between the two principal components of the axially symmetric ^{15}N chemical shift tensor, r_{NH} is the ^{1}H-^{15}N internuclear distance (1.02 x 10^{-10} m, θ is the angle between the ^{15}N chemical shift tensor and the N-H bond vector (17°), and ω_N is the Larmor frequency for ^{15}N. To account for the difference in viscosity between the D_2O-based buffer required for the relaxation violated coherence transfer experiment and the H_2O-based buffer required for the TRACT experiment, the value of τ_c was scaled by 1.45 to account for the increased viscosity of D_2O. This scaling factor was determined by measuring the viscosity of 20% glycerol in D_2O v/v at 20 °C using a Cannon-Fenske viscometer. Then a pulsed field gradient experiment (ledbpgppr2s from Bruker library) with 1000 μs gradient duration at 400 MHz on a Bruker Avance NEO spectrometer was used to determine the temperature dependence of the diffusion coefficient of phenylalanine in the NMR buffer used for the Ileδ1-[^{13}C,^{1}H] samples at 22 °C, 23.5 °C, and 25 °C in a 3 mm NMR tube. The gradient strength was increased in a squared manner from 5% to 95% in 16 steps with 8 scans each. The diffusion coefficient was extrapolated to 20 °C by linear fit and the Stokes-Einstein equation was used to determine the viscosity at 25 °C [2].

The of τ_c determined by TRACT for 3C was 28.7 ± 1.6 ns. The value of τ_c needed so that the highest order parameter is equal to 1 is 24.8 ns, and the value calculated by HydroNMR was 25.1 ns. The value of τ_c from TRACT is within 15%, and the differences in order parameters identified in the main text are large enough for our analysis to hold even with a τ_c for 3C of 28.7 ns and τ_c determined by setting the highest order parameter to equal 1 for 3C*D.

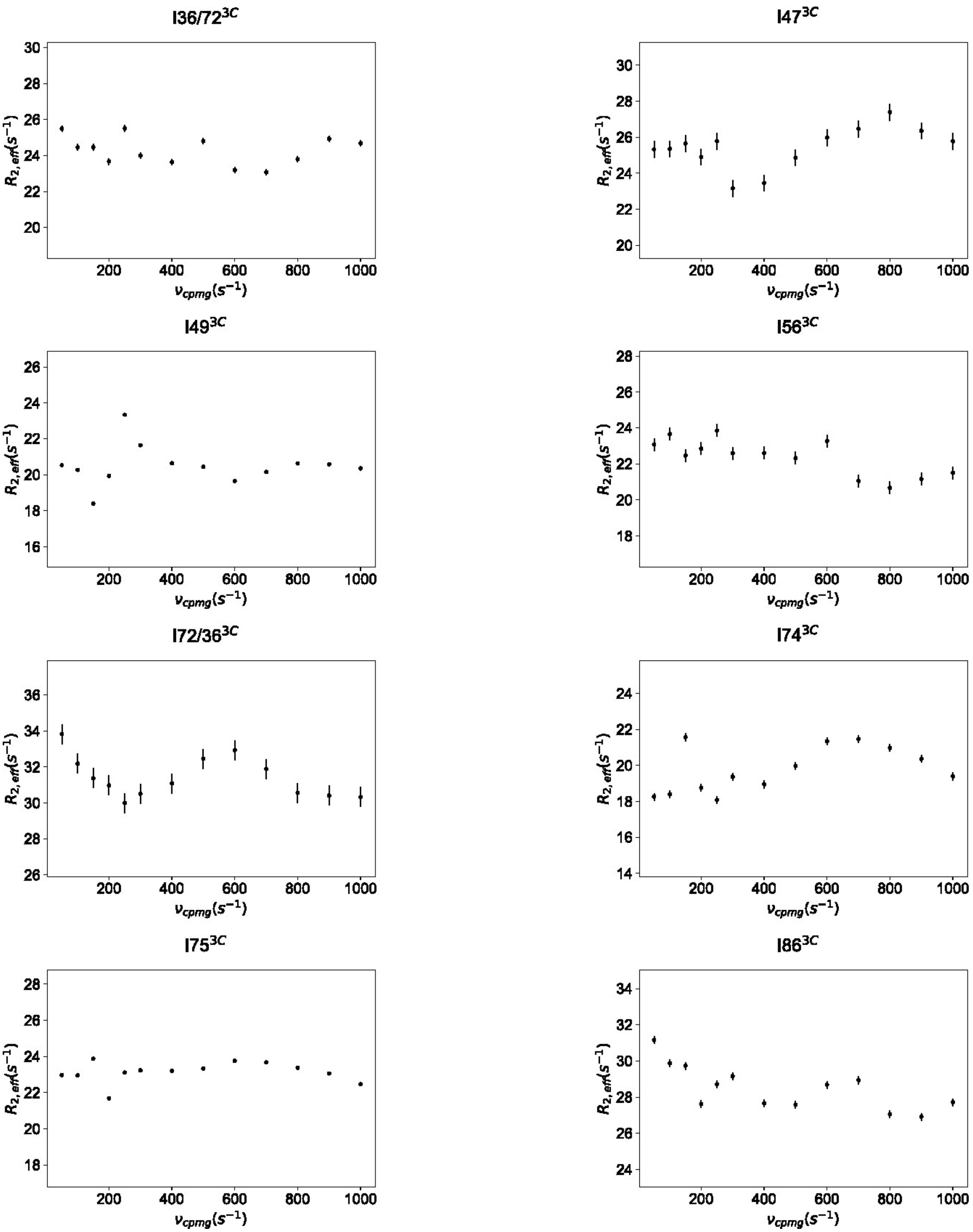

I36/72^{3C}
I47^{3C}
I49^{3C}
I56^{3C}
I72/36^{3C}
I74^{3C}
I75^{3C}
I86^{3C}
$R_{2,eff}(s^{-1})$
$\nu_{cpmg}(s^{-1})$

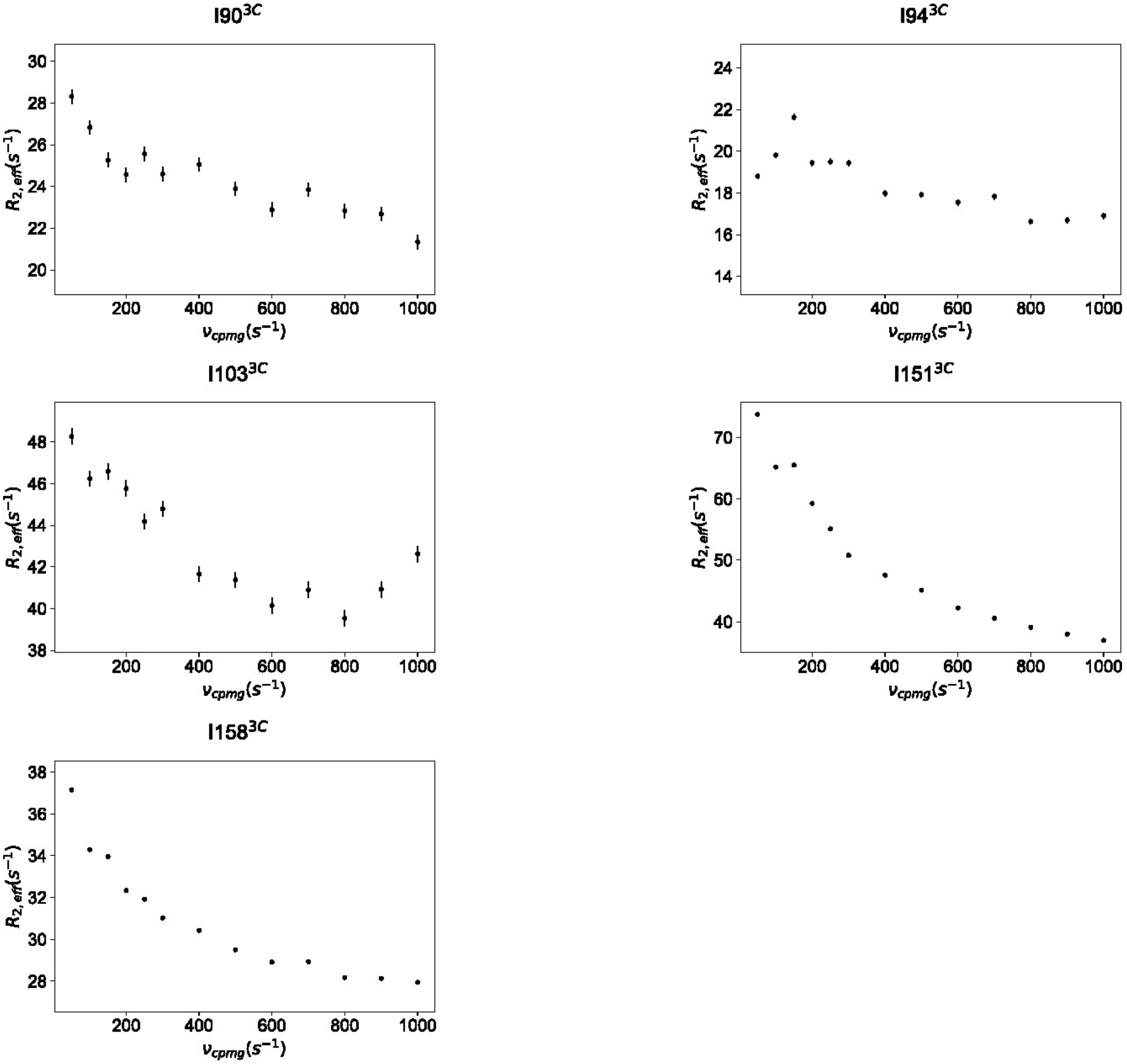

Figure S1. Multiple quantum ^{1}H-^{13}C CPMG relaxation dispersion plots for d1-methyl Ile groups in the 3C protein.

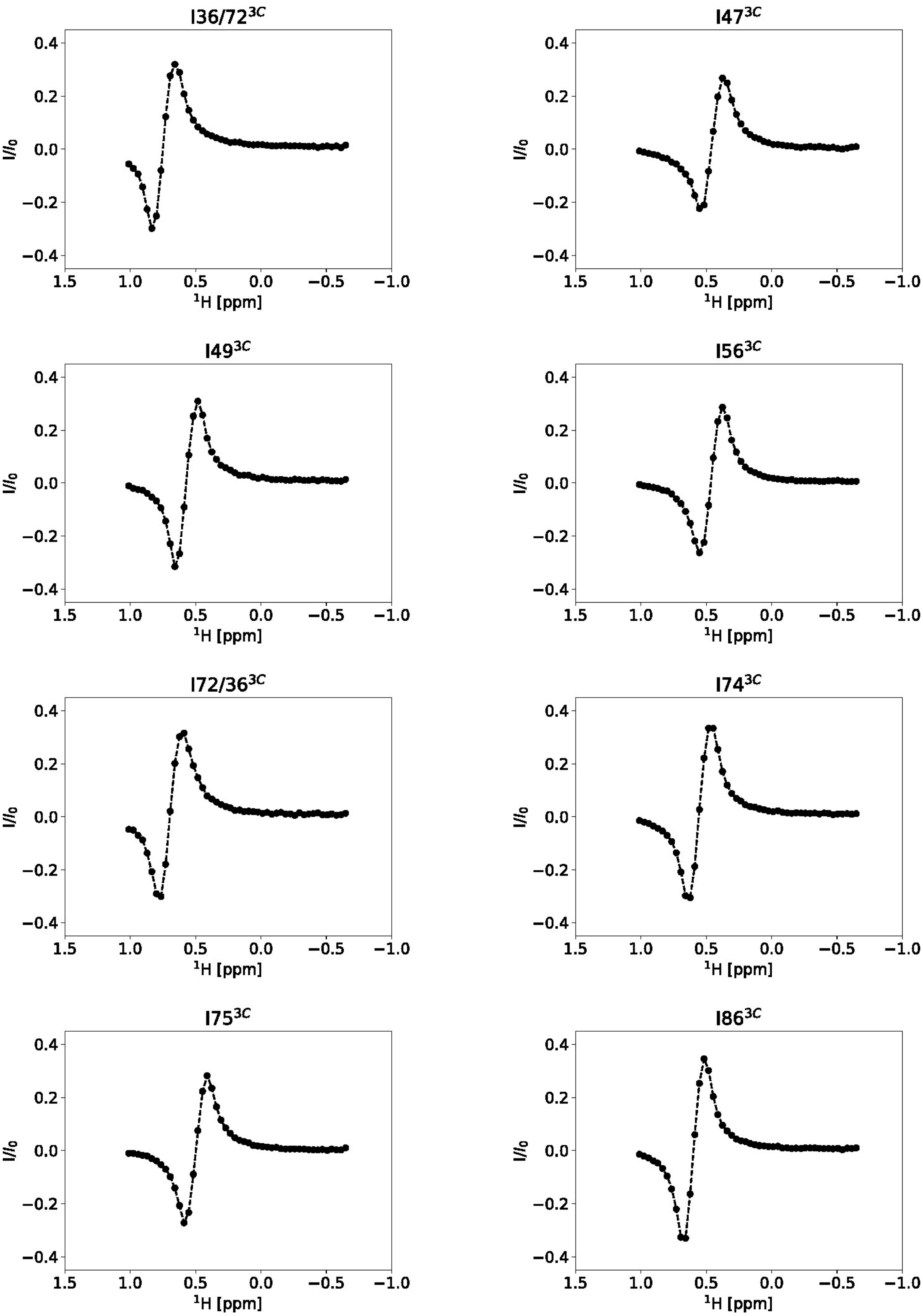
I36/72^{3C}
I47^{3C}
I49^{3C}
I56^{3C}
I72/36^{3C}
I74^{3C}
I75^{3C}
I86^{3C}
I/I_0
^{1}H [ppm]
0.4
0.2
0.0
−0.2
−0.4
1.5
1.0
0.5
0.0
−0.5
−1.0

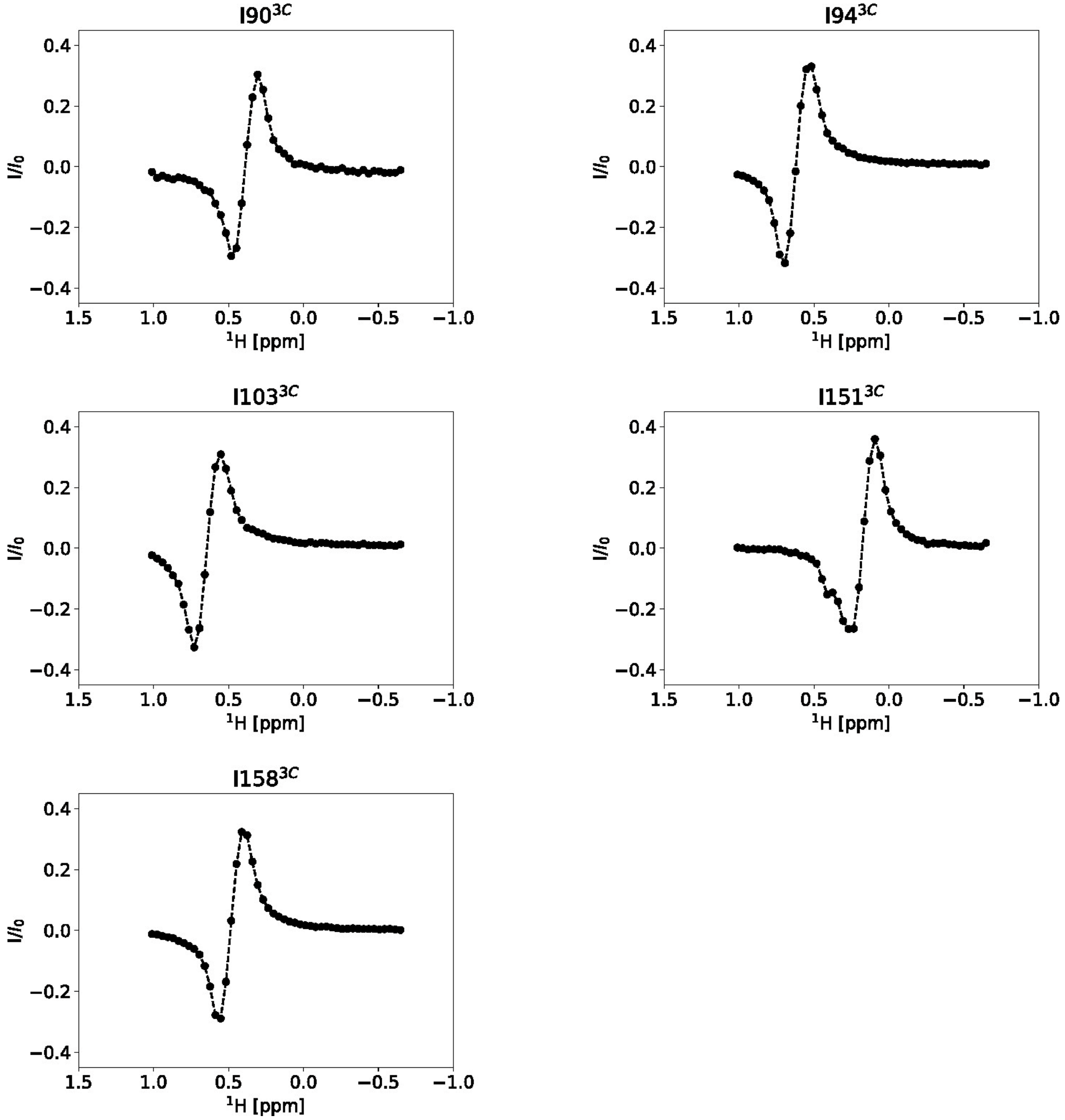

Figure S2. ^{1}H CEST profiles for d1-methyl Ile groups in the 3C protein.

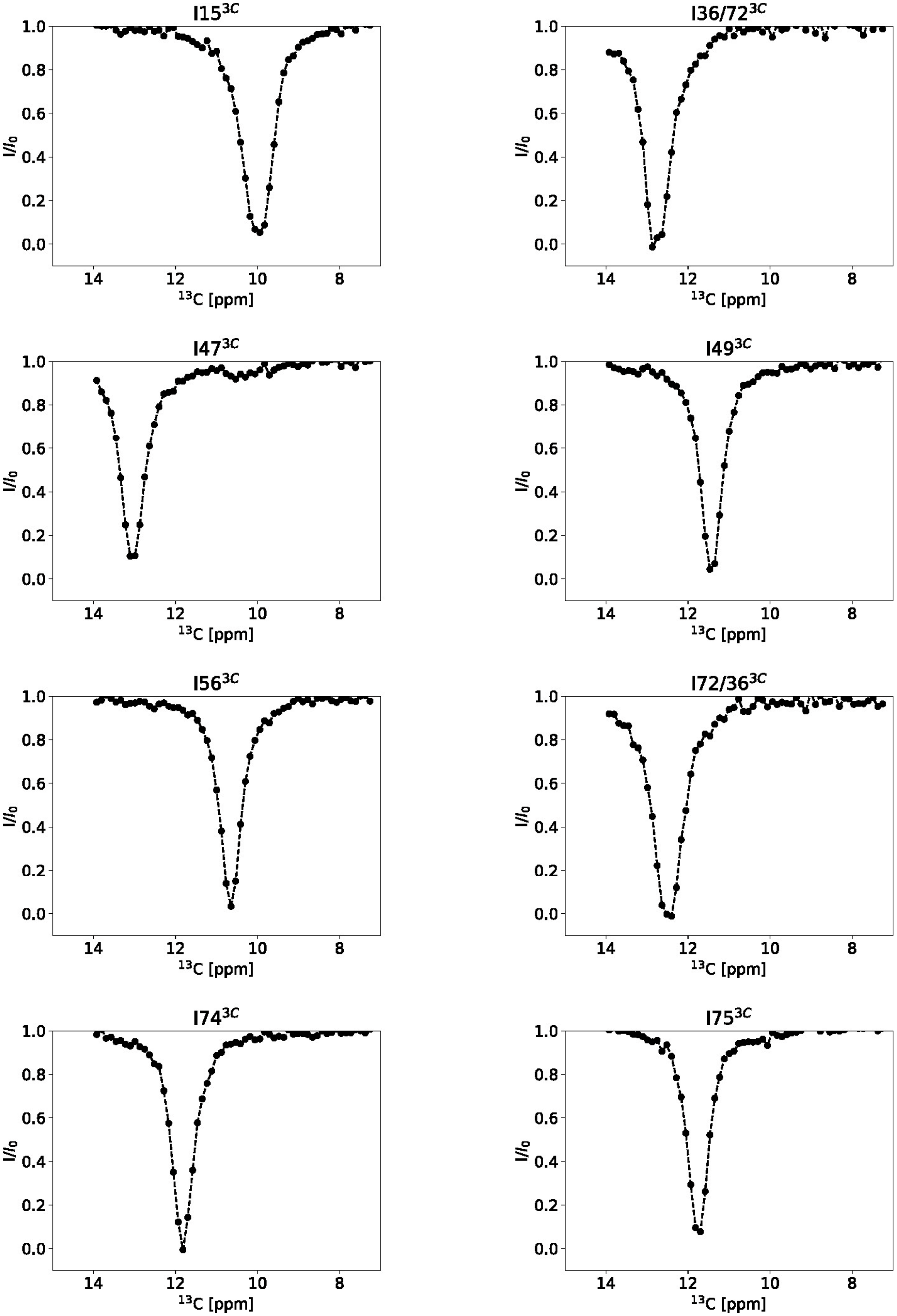

I15^{3C}
I36/72^{3C}
I47^{3C}
I49^{3C}
I56^{3C}
I72/36^{3C}
I74^{3C}
I75^{3C}
I/I$_0$
^{13}C [ppm]

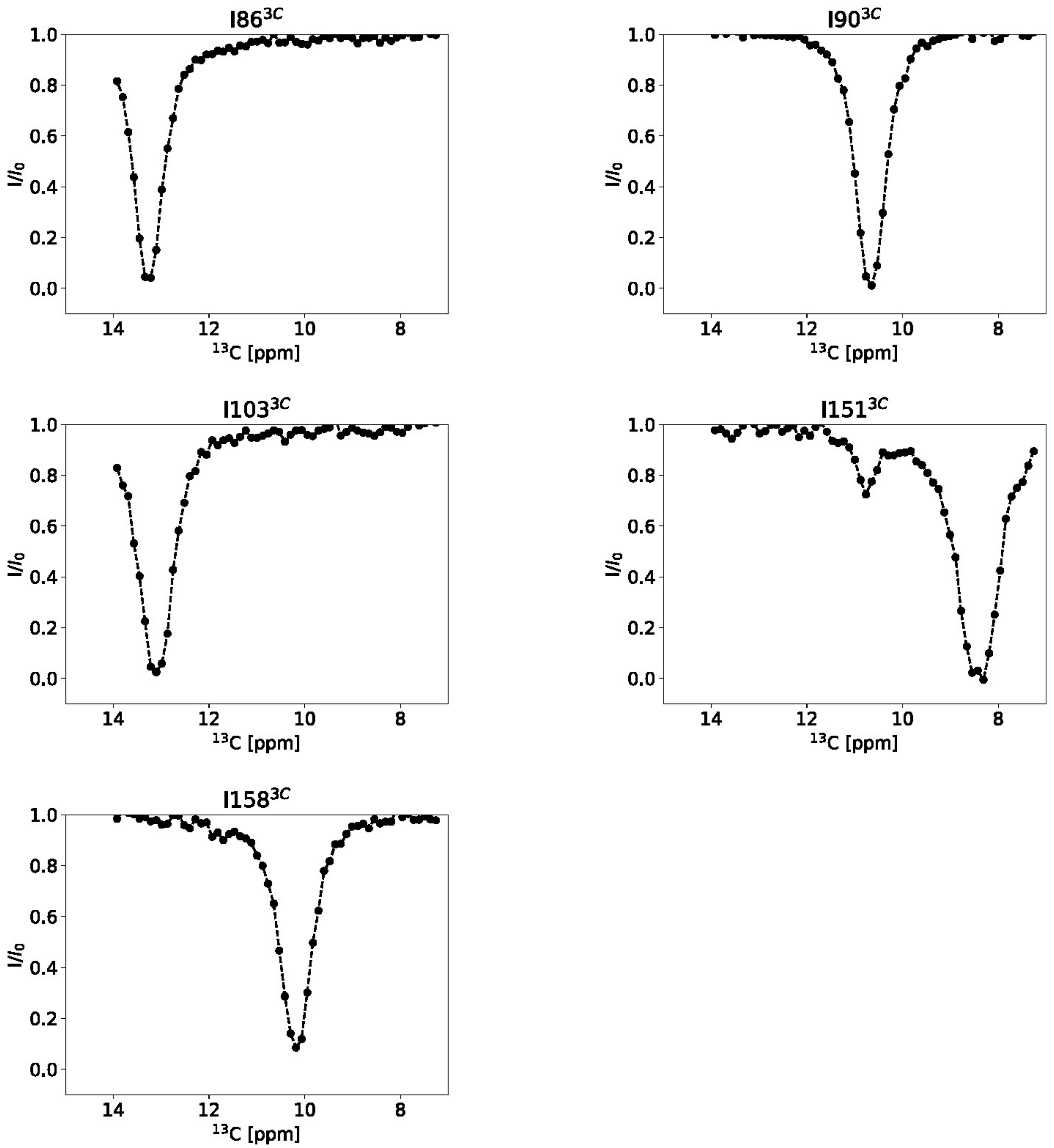

Figure S3. ^{13}C CEST profiles for d1-methyl Ile groups in the 3C protein.

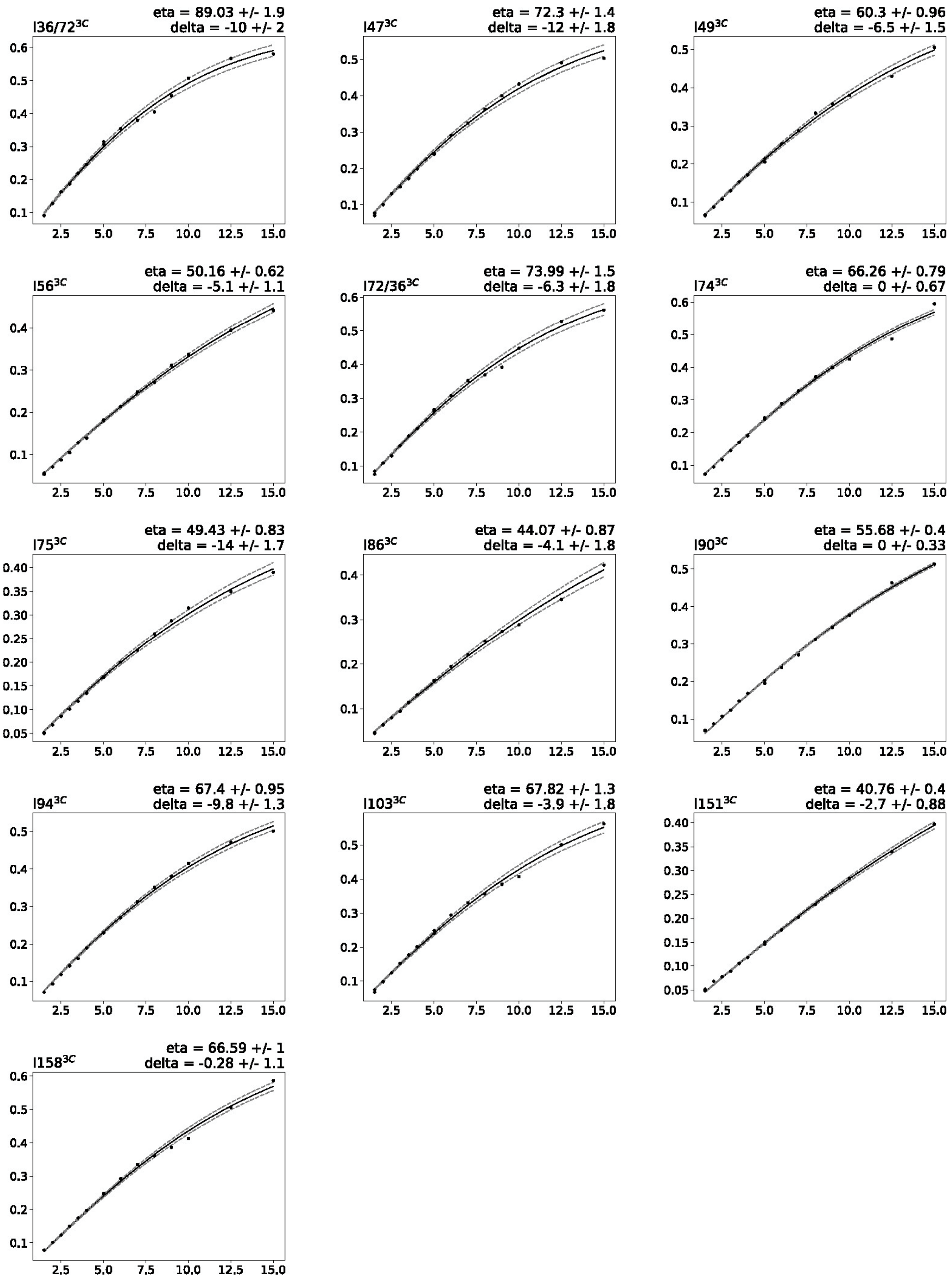

Figure S4. Nonlinear fits for the intramethyl ^{1}H-^{1}H cross-correlated relaxation (eta), including the correction for dipolar interactions with external protons (delta) for d1-methyl Ile groups in the 3C protein. Units for eta and delta are s^{-1}.

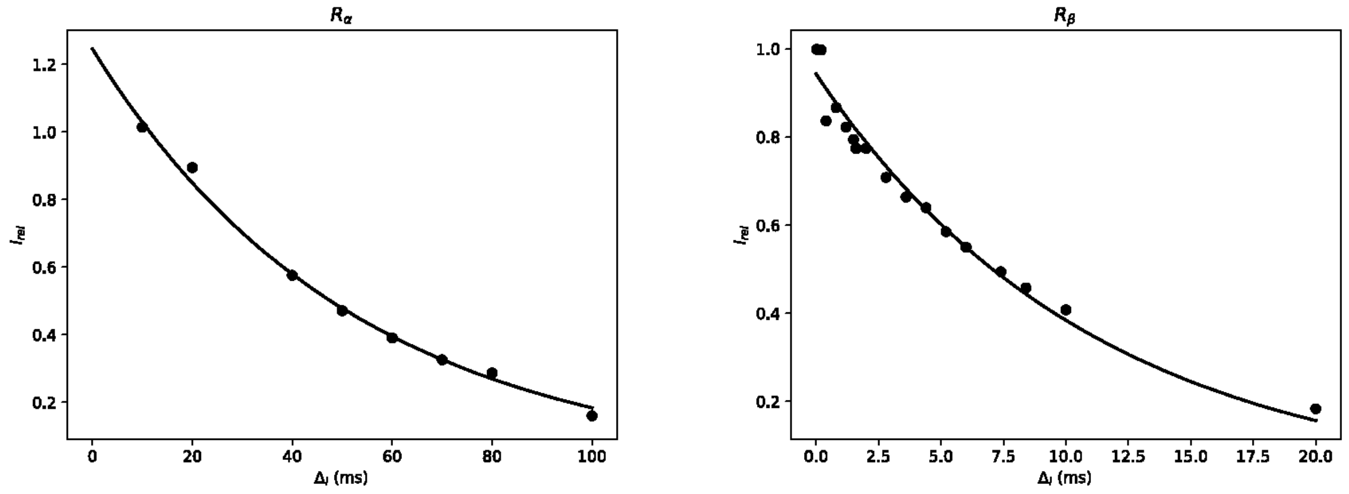

Figure S5. Fitted ^{1}H-^{15}N TRACT results for determination of τ_c for 3C.

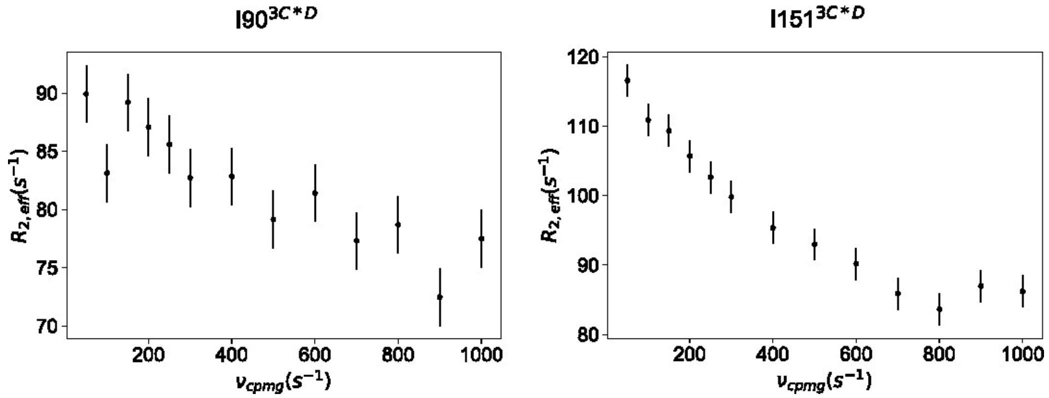

Figure S6. Multiple quantum ^{1}H-^{13}C CPMG relaxation dispersion plots for d1-methyl Ile groups in the 3C domain of the 3CD protein (i.e. 3C*D). Only curves with measurable R_{ex} values are shown; other curves are relatively flat or overly noisy preventing estimation of R_{ex} values.

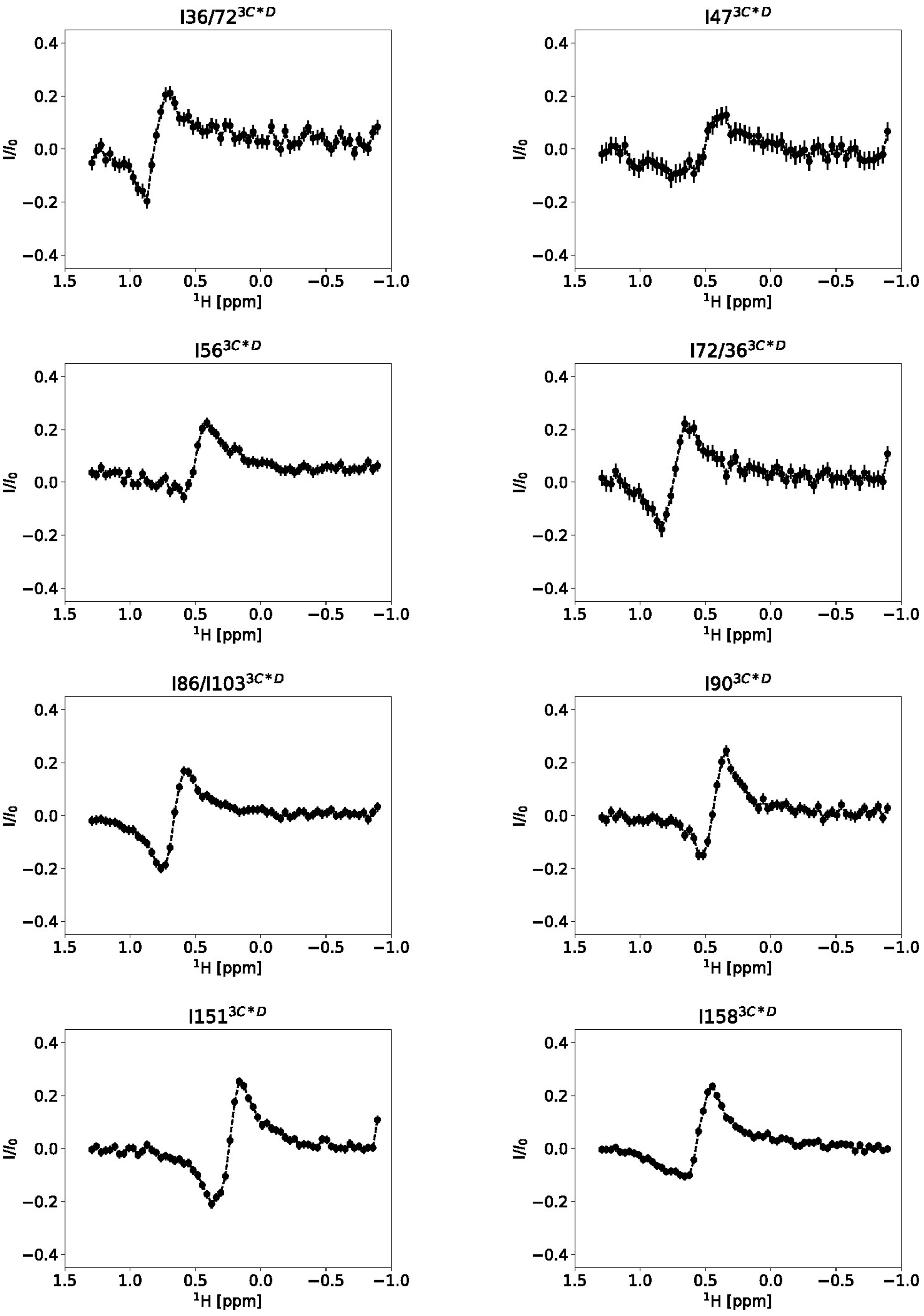

Figure S7. ^{1}H CEST profiles for d1-methyl Ile groups in the 3C domain of the 3CD protein (i.e. 3C*D).

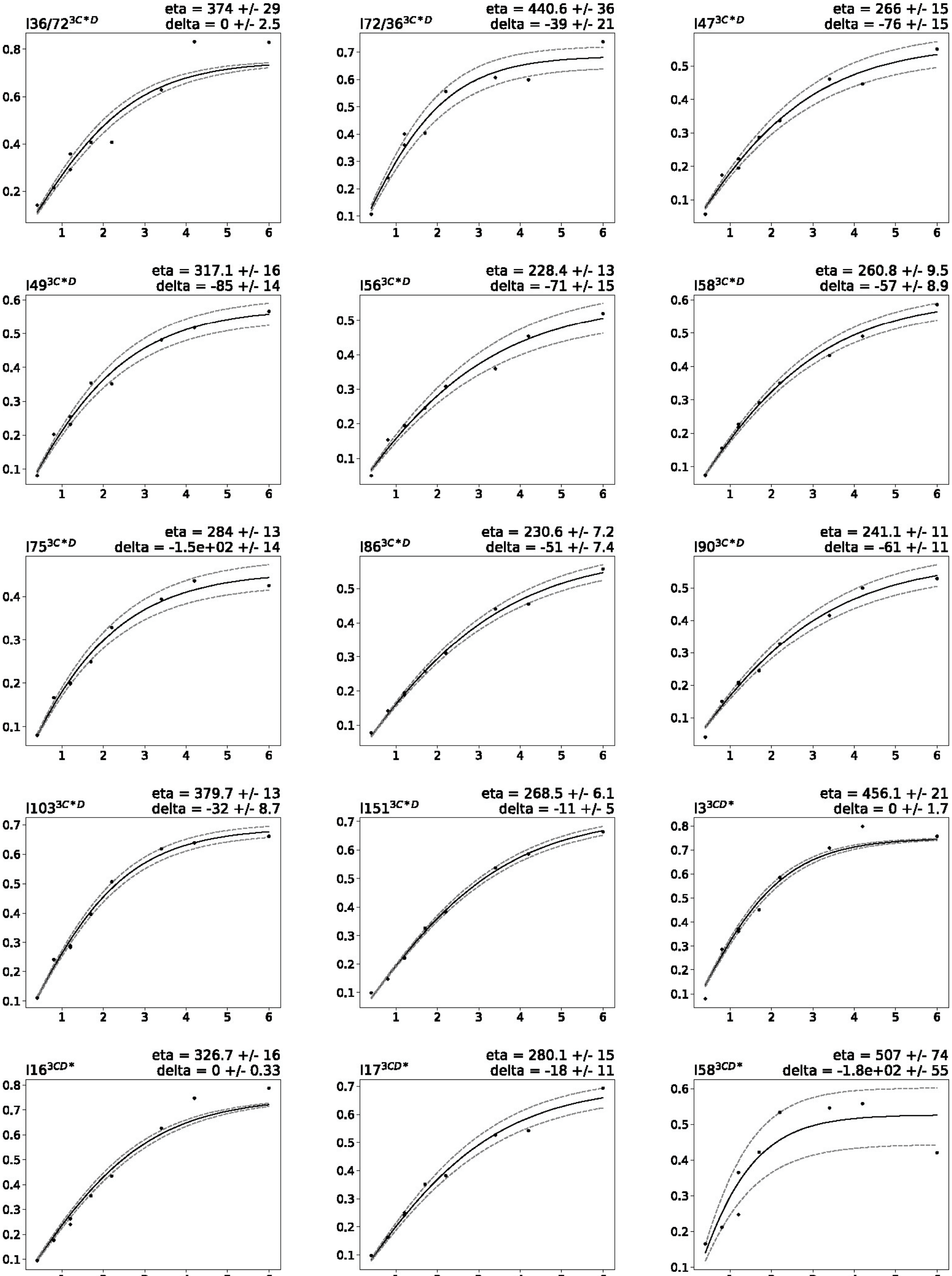
I36/72^{3C*D}
eta = 374 +/- 29
delta = 0 +/- 2.5
I72/36^{3C*D}
eta = 440.6 +/- 36
delta = -39 +/- 21
I47^{3C*D}
eta = 266 +/- 15
delta = -76 +/- 15
I49^{3C*D}
eta = 317.1 +/- 16
delta = -85 +/- 14
I56^{3C*D}
eta = 228.4 +/- 13
delta = -71 +/- 15
I58^{3C*D}
eta = 260.8 +/- 9.5
delta = -57 +/- 8.9
I75^{3C*D}
eta = 284 +/- 13
delta = -1.5e+02 +/- 14
I86^{3C*D}
eta = 230.6 +/- 7.2
delta = -51 +/- 7.4
I90^{3C*D}
eta = 241.1 +/- 11
delta = -61 +/- 11
I103^{3C*D}
eta = 379.7 +/- 13
delta = -32 +/- 8.7
I151^{3C*D}
eta = 268.5 +/- 6.1
delta = -11 +/- 5
I3^{3CD*}
eta = 456.1 +/- 21
delta = 0 +/- 1.7
I16^{3CD*}
eta = 326.7 +/- 16
delta = 0 +/- 0.33
I17^{3CD*}
eta = 280.1 +/- 15
delta = -18 +/- 11
I58^{3CD*}
eta = 507 +/- 74
delta = -1.8e+02 +/- 55

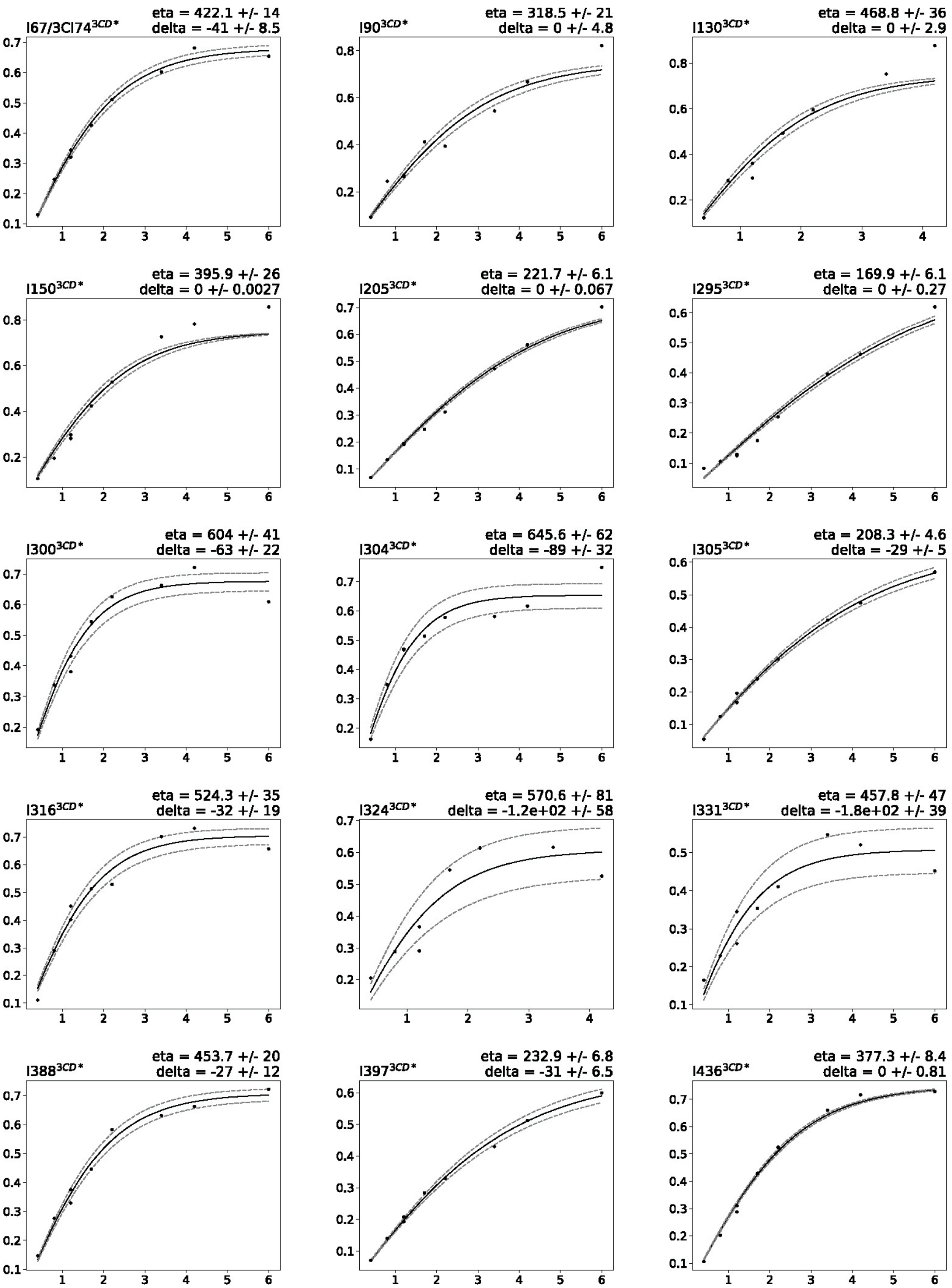

Figure S8. Nonlinear fits for the intramethyl ^{1}H-^{1}H cross-correlated relaxation (eta), including the correction for dipolar interactions with external protons (delta) for d1-methyl Ile groups in the 3CD protein. Units for eta and delta are s^{-1}. 3C*D and 3CD* indicate Ile residues in the 3C and 3D domains, respectively, such that the 3CD* numbering follows the 3D numbering convention.

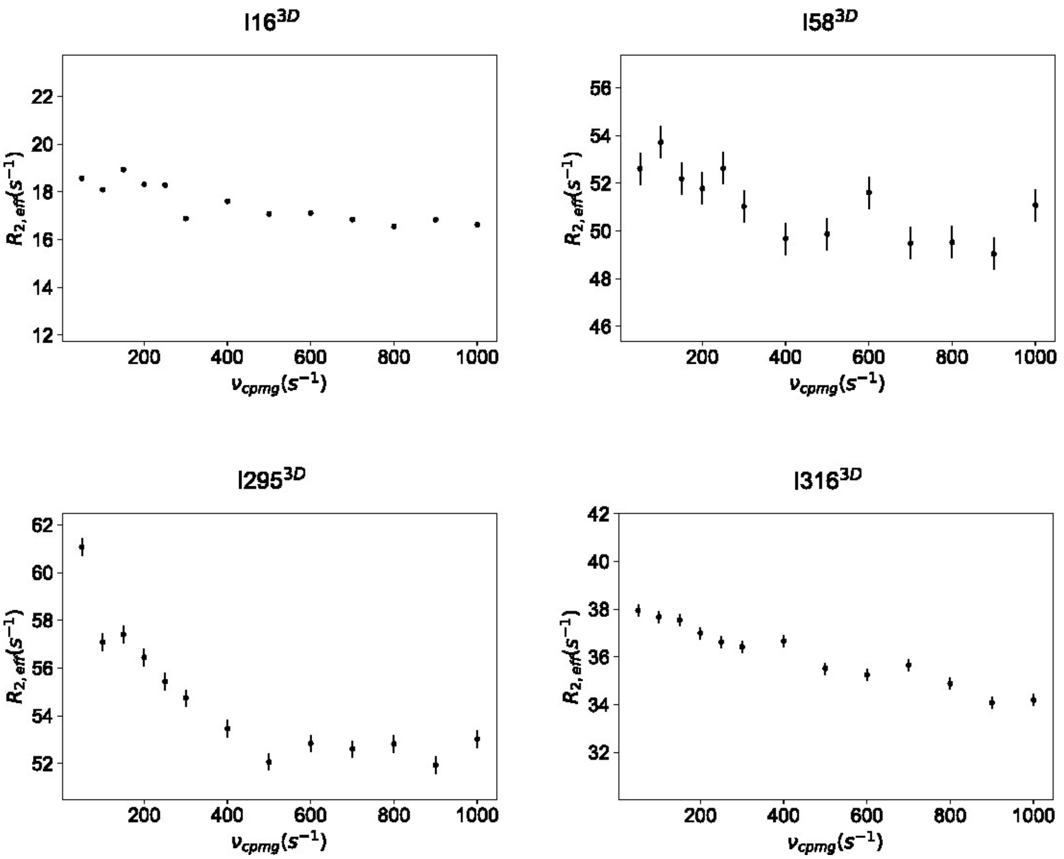

Figure S9. Multiple quantum ^{1}H-^{13}C CPMG relaxation dispersion plots for d1-methyl Ile groups in 3D protein. Only curves with measurable R_{ex} values are shown; other curves are relatively flat or overly noisy preventing estimation of R_{ex} values.

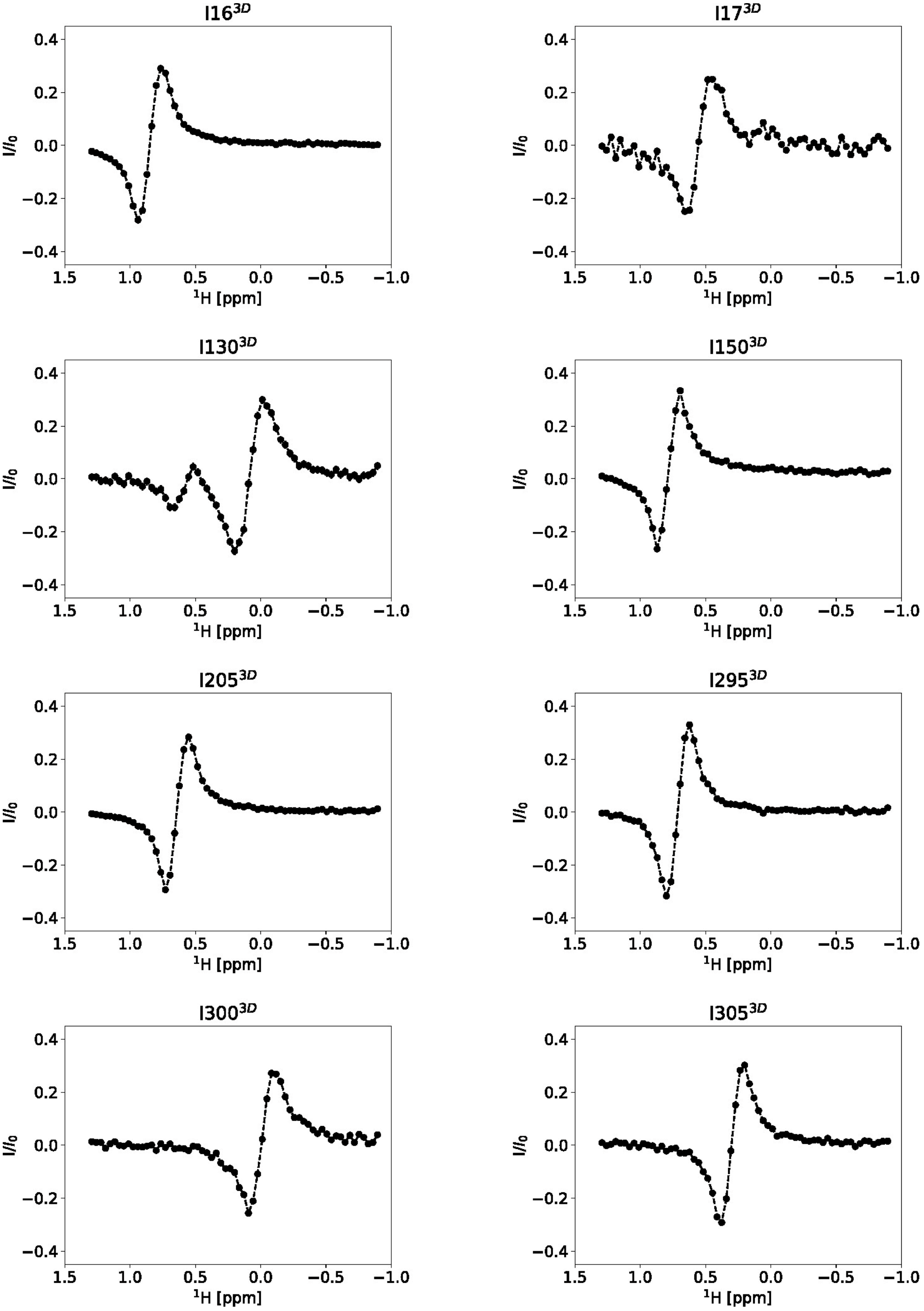
I16^{3D}
I17^{3D}
I130^{3D}
I150^{3D}
I205^{3D}
I295^{3D}
I300^{3D}
I305^{3D}
I/I_0
^{1}H [ppm]
0.4
0.2
0.0
−0.2
−0.4
1.5
1.0
0.5
0.0
−0.5
−1.0

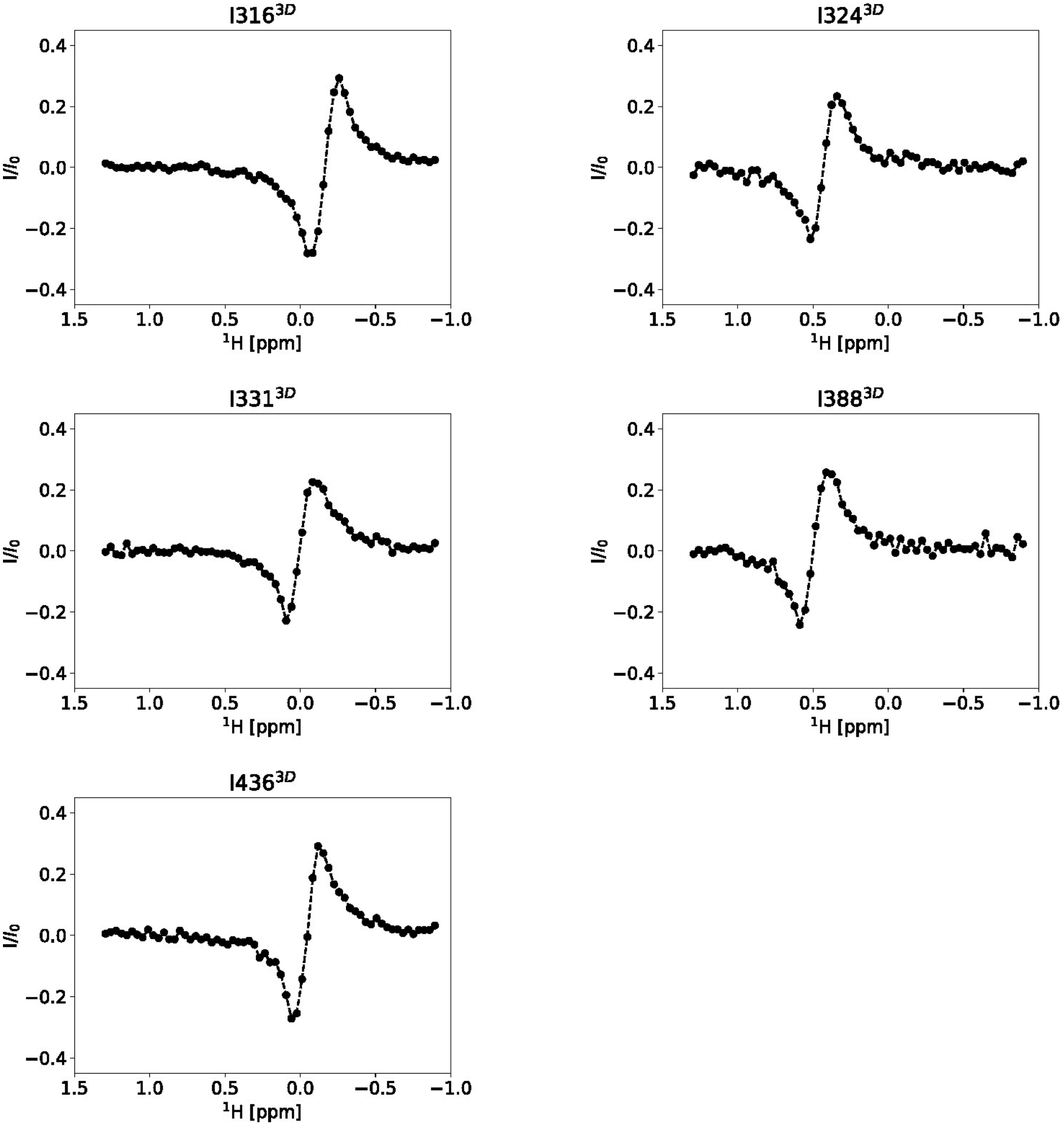

Figure S10. ^{1}H CEST profiles for d1-methyl Ile groups in the 3D protein.

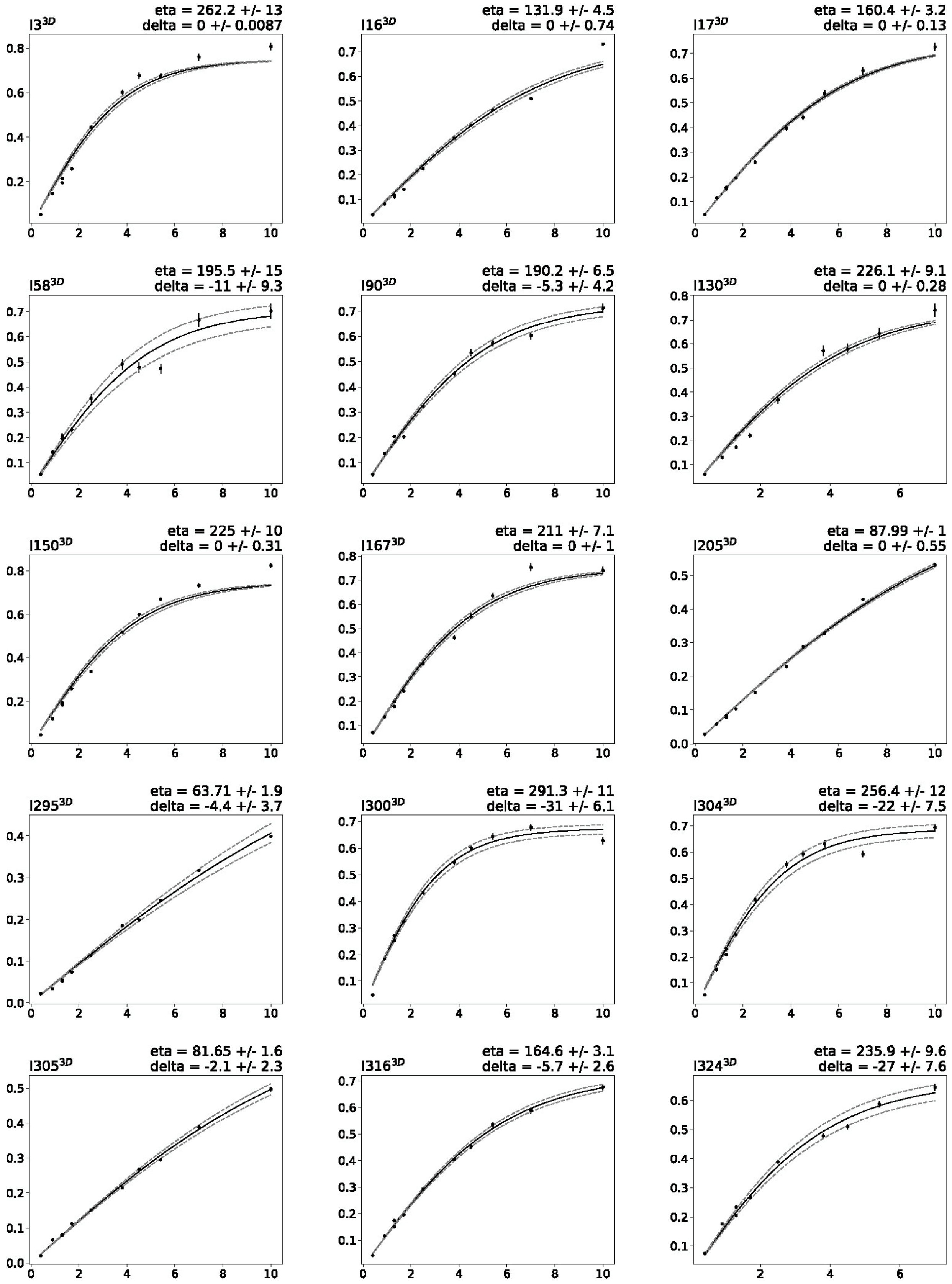
I3^{3D}
eta = 262.2 +/- 13
delta = 0 +/- 0.0087
I16^{3D}
eta = 131.9 +/- 4.5
delta = 0 +/- 0.74
I17^{3D}
eta = 160.4 +/- 3.2
delta = 0 +/- 0.13
I58^{3D}
eta = 195.5 +/- 15
delta = -11 +/- 9.3
I90^{3D}
eta = 190.2 +/- 6.5
delta = -5.3 +/- 4.2
I130^{3D}
eta = 226.1 +/- 9.1
delta = 0 +/- 0.28
I150^{3D}
eta = 225 +/- 10
delta = 0 +/- 0.31
I167^{3D}
eta = 211 +/- 7.1
delta = 0 +/- 1
I205^{3D}
eta = 87.99 +/- 1
delta = 0 +/- 0.55
I295^{3D}
eta = 63.71 +/- 1.9
delta = -4.4 +/- 3.7
I300^{3D}
eta = 291.3 +/- 11
delta = -31 +/- 6.1
I304^{3D}
eta = 256.4 +/- 12
delta = -22 +/- 7.5
I305^{3D}
eta = 81.65 +/- 1.6
delta = -2.1 +/- 2.3
I316^{3D}
eta = 164.6 +/- 3.1
delta = -5.7 +/- 2.6
I324^{3D}
eta = 235.9 +/- 9.6
delta = -27 +/- 7.6

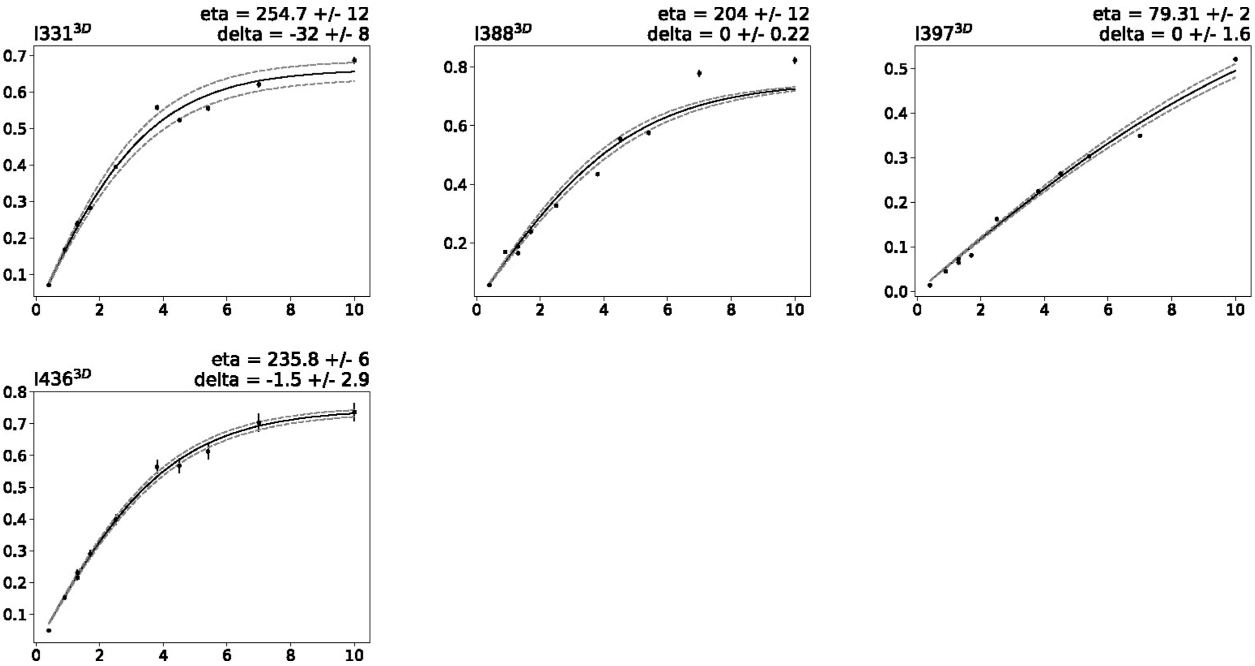

Figure S11. Nonlinear fits for the intramethyl ^{1}H-^{1}H cross-correlated relaxation (eta), including the correction for dipolar interactions with external protons (delta) for d1-methyl Ile groups in the 3D protein. Units for eta and delta are s^{-1}.

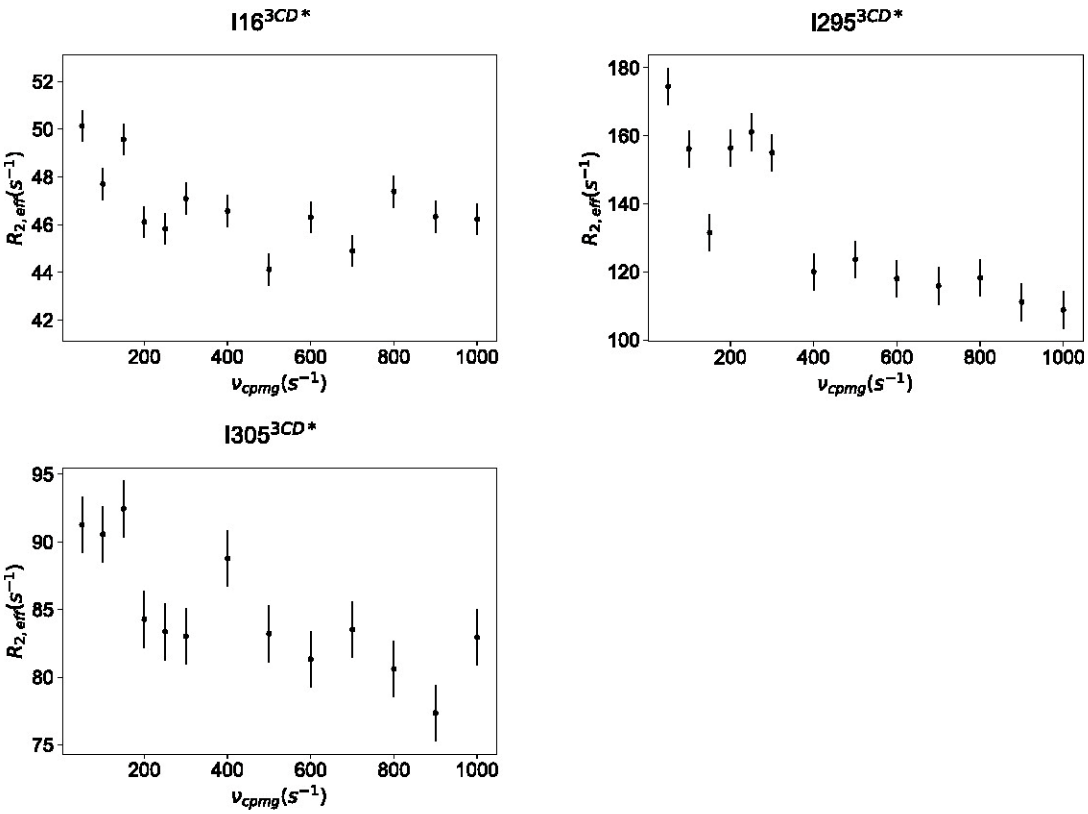

Figure S12. Multiple quantum ^{1}H-^{13}C CPMG relaxation dispersion plots for d1-methyl Ile groups in the 3D domain of the 3CD protein (i.e. 3CD*). Only curves with measurable R_{ex} values are shown; other curves are relatively flat or overly noisy preventing estimation of R_{ex} values.

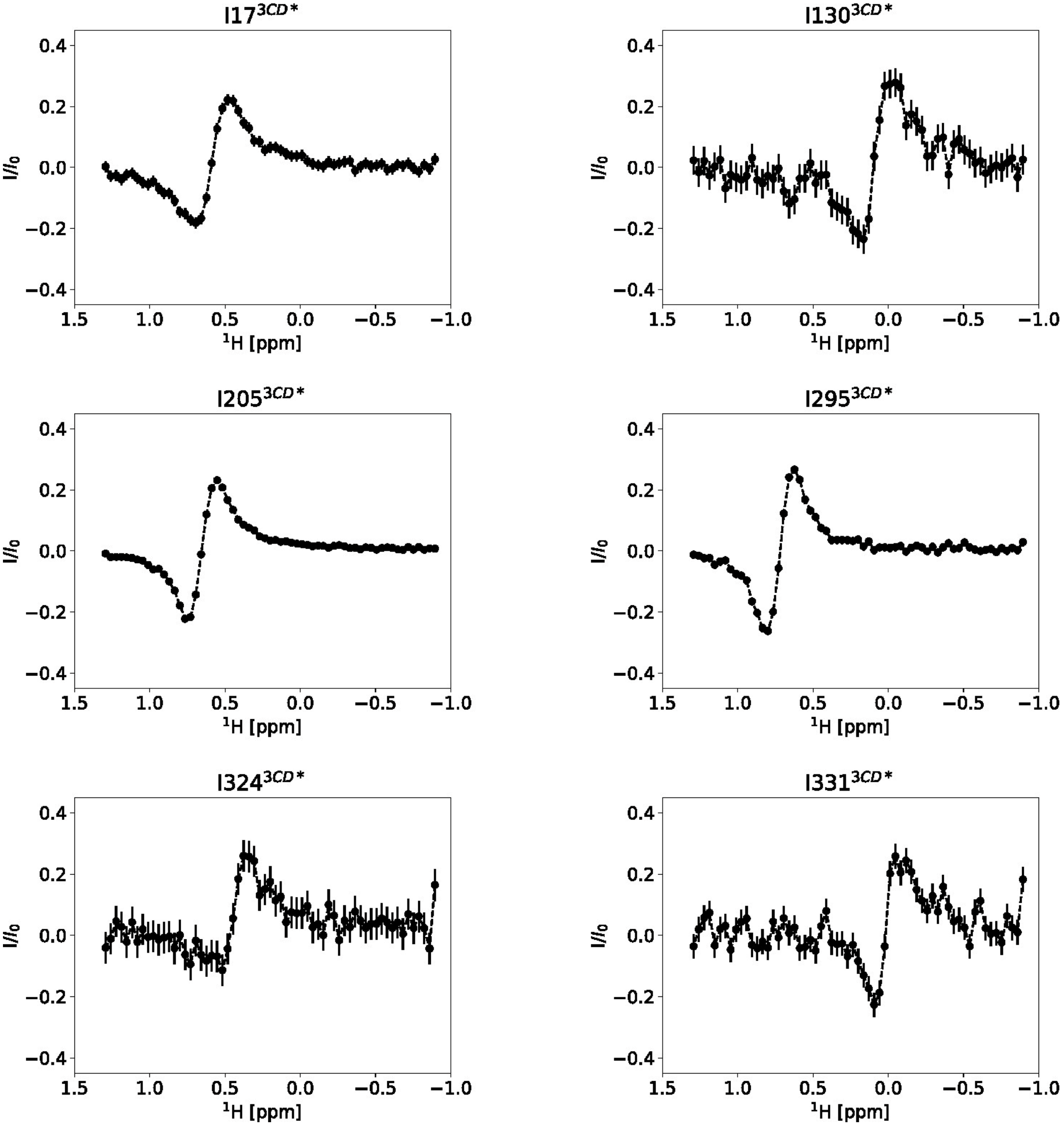
I17^{3CD*}
I130^{3CD*}
I205^{3CD*}
I295^{3CD*}
I324^{3CD*}
I331^{3CD*}
I/I_0
^{1}H [ppm]
0.4
0.2
0.0
−0.2
−0.4
1.5
1.0
0.5
0.0
−0.5
−1.0

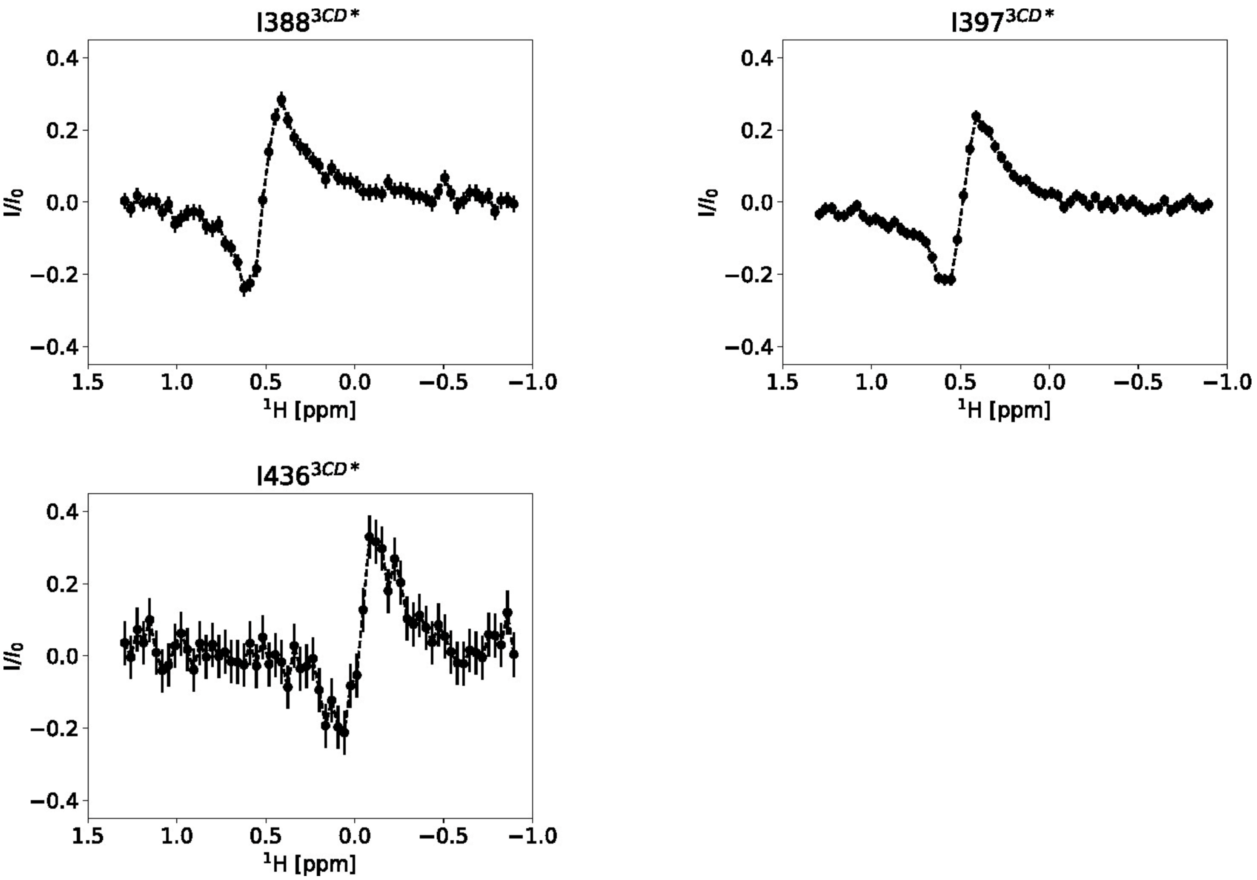

Figure S13. ^{1}H CEST profiles for d1-methyl Ile groups in the 3D domain of the 3CD protein (i.e. 3CD*).

References

1. Lee, D.; Hilty, C.; Wider, G.; Wüthrich, K. Effective Rotational Correlation Times of Proteins from NMR Relaxation Interference. *J. Magn. Reson.*, **2006**, *178*, 72–76.

2. Li, W.; Kagan, G.; Hopson, R.; Williard, P.G. Measurement of Solution Viscosity via Diffusion-Ordered NMR Spectroscopy (DOSY). *J. Chem. Educ.*, **2011**, *88*, 1331–1335
